# Accumulative Difference Image Protocol for Particle Tracking in Fluorescence Microscopy Tested in Mouse Lymphonodes

**DOI:** 10.1371/journal.pone.0012216

**Published:** 2010-08-17

**Authors:** Carlo E. Villa, Michele Caccia, Laura Sironi, Laura D'Alfonso, Maddalena Collini, Ilaria Rivolta, Giuseppe Miserocchi, Tatiana Gorletta, Ivan Zanoni, Francesca Granucci, Giuseppe Chirico

**Affiliations:** 1 Dipartimento di Fisica, Università degli Studi di Milano Bicocca, Milano, Italy; 2 Dipartimento di Medicina Sperimentale, Università degli Studi di Milano Bicocca, Monza, Italy; 3 Dipartimento di Biotecnologie e Bioscienze, Università degli Studi di Milano Bicocca, Milano, Italy; University of New Mexico, United States of America

## Abstract

The basic research in cell biology and in medical sciences makes large use of imaging tools mainly based on confocal fluorescence and, more recently, on non-linear excitation microscopy. Substantially the aim is the recognition of selected targets in the image and their tracking in time. We have developed a particle tracking algorithm optimized for low signal/noise images with a minimum set of requirements on the target size and with no a priori knowledge of the type of motion. The image segmentation, based on a combination of size sensitive filters, does not rely on edge detection and is tailored for targets acquired at low resolution as in most of the in-vivo studies. The particle tracking is performed by building, from a stack of Accumulative Difference Images, a single 2D image in which the motion of the whole set of the particles is coded in time by a color level. This algorithm, tested here on solid-lipid nanoparticles diffusing within cells and on lymphocytes diffusing in lymphonodes, appears to be particularly useful for the cellular and the in-vivo microscopy image processing in which few a priori assumption on the type, the extent and the variability of particle motions, can be done.

## Introduction

Fluorescence Microscopy with linear and non linear excitation allows to follow cells within tissues and nanoparticles within the cell cytoplasm [1], [2]. Manual tracking of particles in a time series of live cell images is a common approach to study the kinetics and the dynamics of cellular and intracellular interactions. This technique is extremely time-consuming and it is prone to some systematic errors. A large effort is then currently under way to develop efficient algorithms for the automatic detection of cells/particles with a minimum action from the user [3]–[8]. Since a complete automatic detection is rarely feasible, we would like to develop general discrimination and tracking methods based on minimum a priori requirements on the shape and the size of the objects, followed by a close inspection of the results made by the user on a compact, though complete, description (2D image) of the trajectories. This is the main aim of the research reported here.

Several algorithms have been devised in the past to detect and follow in time macroscopic objects on sonar, radar or astronomical images. They have been also applied to ultrasound images of human tissues and to the analysis of the microscopic characterization of biological specimens [9]. The first algorithms developed in the '60s for military purposes [9] were adapted to velocimetry [10] and later gave the input to the first fluorescent particle tracking methods [11]. These algorithms lost much of their power when adapted to other studies since any assumption of a definite shape or physical model for the object's movements (for example, minimum curvature radius, acceleration/deceleration range) cannot be easily applied to the biological field even though relevant advances in this direction have been recently reported [7], [12].

The tracking algorithms are always based on two steps: segmentation, that aims to detect objects on each image of a time series, and the actual tracking, that is devoted to follow the objects in time on a sequential series of images. Required features are the reliability of the image segmentation, the low number of false positive assignments, the low number of mis-tracking (particularly relevant for images with high density and interacting objects) and fast tracking times. Not all these requirements can be simultaneously fulfilled and a compromise must be found for the specific case under study. Most of the existing tracking codes have been recently reviewed and tested on high signal to noise images [12]. The codes reviewed are based mainly on the threshold and the edge detection segmentation algorithms [13]. The specific algorithm proposed by Hand et al. [12] is based on the watershed segmentation method [13], that allows to detect also interacting targets, and the target tracking is obtained by image registration [14]. This method is based on the assumption that the target positions in subsequent images can be converted one into the other only by translation/rotation/stretching of the frames. Regularization requirements on the deformation of the objects to track (evolving neural stem cells for example) and assumptions on the stochastic properties of their motion have been applied also in other works such as the one reported recently by the Degerman's group [7]. The methods developed in [7] and based on the seminal work by Chan and Vese [15], are extremely powerful for the cases in which details on the shape of the targets and their deformation in time must be retrieved. The widely used SpotTracker algorithm [16], makes instead use of prefiltering of the images (Mexican Hat filter) to enhance the signal/noise ratio and it is therefore particularly suited for highly noisy images. The method adopted by Sage et al. [16] makes a careful analysis of the noise on the fluorescence image but stands then on the assumption that only one target per frame must be retrieved. All these types of assumptions seem not to be particularly suited for the analysis of images taken on dense (more than one particle to be tracked per frame) and low signal/noise sample of particles whose motion does not fulfill any defined regularization property (average step length or deviation angle per frame, etc.). Moreover a parallel visualization method to treat a whole bunch of trajectories, possibly on a single 2D image, would greatly help the user in the biophysical analysis of the experiments.

The conditions and the requirements described above are typical of a number of biomedical research areas. We focus in this report on two such cases that play the role of examples of two relevant issues in biomedical and pharmaceutical studies: the interactions of nanoparticles (NPs) with cells and of their intracellular (Brownian) motion, and the investigation of cell dynamics and mutual interaction within tissues in immunology. Despite the fact that fluorescent markers provide a high number of photons per frame and would in this sense offer high signal/noise images, a number of undesired signals arise from the NP trapping matrix, cell cytoplasm or tissue [17]. Auto-fluorescence is typically the most effective in raising the background level on the image especially because it is easily primed in the visible (around 500 nm, due to Flavin enzymes) and in the near infrared with a broad excitation band [18]. The use of nonlinear excitation is in fact particularly important in in-vivo imaging studies where one wants to follow fluorescently labeled cells in tissues. An example is given by the study of the interactions between lymphocytes within the lymphoid organs, such as that engaged within the framework of the EU project ENCITE (http://www.encite.org). In the case of NPs carrying drugs and/or fluorescent markers, on the other hand, undesired signals that lower the signal/noise ratio may arise also from the presence of unloaded or released markers.

The examples chosen here are particularly suited also to illustrate some of the problems connected with the use of regularization criteria for the motion of particles or cells. For the example, the lymphocytes undergo an activation process, aided by the interaction with dendritic cells [19], that involves many short and long living contacts between lymphocytes. These events are difficult to discern automatically on the images, even when different staining is used for the various types of lymphocytes, due to the presence of cross-talk between the image channels.

We are then interested in devising a specific algorithm that can provide us with a batch of segmented targets that fulfill a minimal requirement on the target size (or volume) and with the corresponding trajectories displayed on a single 2D image for a rapid and efficient user analysis. The algorithm that we present and test here is based on the use of image filters that assume only the average size of the targets, on the computation of a time series of Accumulative Differential Images (ADI) and on the construction from these and for each of the targets segmented on the first image of the series, of a single 2D image in which the color of the pixels codes for its passage time through the pixel.

## Materials and Methods

### Two-photon setup

The optical setup was built around a confocal scanning head (FV-300, Olympus, Japan) mounted on an upright optical microscope (BX51, Olympus, Japan) equipped with a high working distance objective (N.A. = 0.95, wd = 2 mm, 20×, water immersion, XLUMPlan FI, Olympus, Japan). The laser source for two-photon excitation was a mode-locked Ti:sapphire laser (Mai Tai HP, Spectra Physics, CA). The images were collected either in descanned, through the FV300 scanning head, or via a custom made non-descanned head [20].

### Lymphocyte extraction and labelling

Transgenic mice (BalbC mice) with DCs expressing wt-GFP were injected with 3–4 millions of labeled Natural Killer (NK) or T cells approximately 24 hours before the experiment. The mouse lymph nodes were extracted from experimental animal and immediately fixed at the bottom of a Petri dish filled with physiological solution at 37°C. During the entire duration of the experiment the temperature inside the box was maintained at 37°C and the physiological solution surrounding the lymph nodes was continuously replaced by “fresh” solution at 37°C saturated with a mixture of 95% O_2_ 5% CO_2_ in order to keep the samples in in vivo conditions. We acquired images on planes in the sub-capsular region between 50 and 200 µm within the lymphonodes for a total volume of 256×256×10 pixels (step sizes Δx = Δy = 1.1–2.76 µm and Δz = 5 µm) and a total acquisition time = 16–35 s. The excitation wavelength was set to λ_TPE_ = 850 nm. We typically followed the motility of the lymphocytes for 60–90 minutes.

### Ethic Statement

We declare that all experiments were performed using protocols approved by the University of Milano-Bicocca Animal Care and Use Committee (also in agreement with the European rules, 86/609/EEG and with the International Guiding Principles for Biomedical Research Involving Animals, as developed by the Council Organizations of Medical Sciences and the Guide for the Care and Use of Laboratory Animals; http://ec.europa.eu/environment/chemicals/lab_animals/revision_en.htm). Mice werehoused in containment facilities of the animal facility and maintained on a regular 12∶12 hour light∶dark cycle with food and water ad libitum.

### Solid Lipid Nanoparticles and Cell Cultures

Solid Lipid Nanoparticles (SLNs) were a kind gift of NANOVECTOR. The main component of the matrix of these particles is tripalmitin (TPM) and SLN are conjugated with 3-(2-Benzothazolyl)-7-(Diethylamino)coumarin (MW 146 D). A30 cells were grown on Petri dishes in DMEM medium supplied with 10% Fetal Bovine Serum (FBS), 1% of L-Glutamine and 1% of Penicillin/Streptomycin and incubated in a controlled environment at 37°C with 5% CO_2_. During experiments cells were incubated with medium supplemented with 1% FBS to prevent NPs aggregation. The images were 256×256 pixels in size and were acquired at a frequency of 0.85 s per frame.

## Results and Discussion

### Particle Tracking Algorithm

The algorithm is composed of a segmentation and a tracking step. The implementation of the algorithm has been made here on the MATLAB platform (with Image Processing Toolbox, by MathWorks, Inc.) but it could be easily translated to other platforms. All the routines and the procedures developed to analyze a time stack of images and to display the results are described in detail in the Supporting Information S1. The MATLAB sources (Matlab, R2009a) are also uploaded as Supporting Information (Code S1). The algorithm is divided in ten steps coded by corresponding routines identified by an initial capital letters from A to K in the routine name. Two sample copies of stack of images collected on Solid Lipid Nanoparticels in cells and on lymphocytes in lymphonodes can be downloaded from the site (http://moby.mib.infn.it/~chirico/tracking.html) for testing the procedures.

### Segmentation

**Figure 1:**
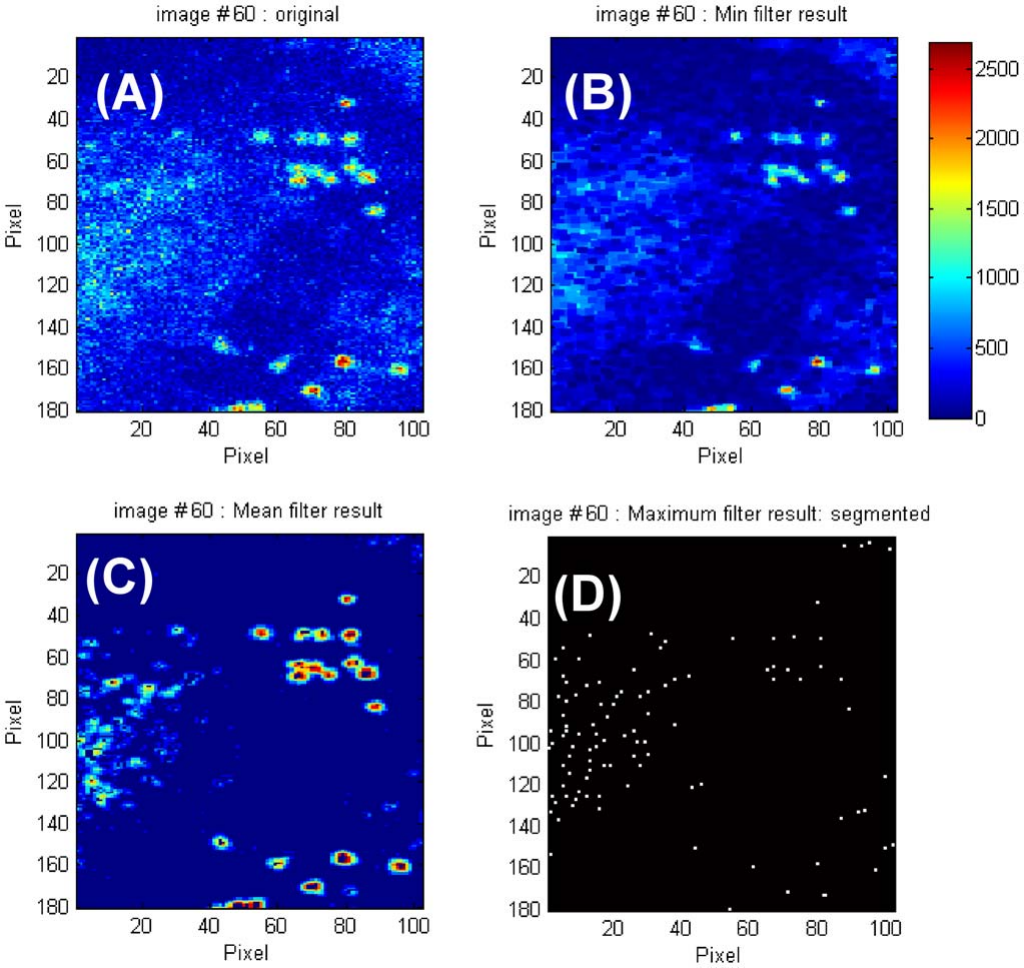
SLNs in epithelial cells. The original image (256×256 pixels, 100×100 µm^2^) of SLNs interacting with epithelial cells is reported in panel A. This image is subject to the minimum filter (panel B) and a threshold filter followed by the mean filter (panel C). All filter matrices (except the threshold one) are based on a 3×3 matrix. Finally a maximum filter is applied to the image in order to reduce each target to single pixels (panel D). In this panel the single pixels that corresponds to the position of the targets have been enlarged to 2×2 submatrices for display purposes. The jet colormap used for the images in panels A to C is reported in the figure.

The segmentation procedure (phase A, routine: *A_Imagefilters.m*) consists of the application of four consecutive filters and provides a binary image in which single saturated pixels correspond to the discerned target positions. The action of the filtering sequence is illustrated on an image (**Fig. 1A**) taken on epithelial cells during the uptake of SLNs. At first a minimum spatial filter [13] (which applies to the central pixel of a (2*m_m_+1*)×(*2m_m_+1*) mask matrix its minimum value) reduces high frequency background components (**Fig. 1B**), according to the procedure:
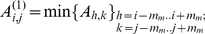
(1)


An outer frame of the size of *m_m_* pixels in each image 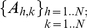 of the stack is excluded from the analysis. In the example treated in **Fig. 1**, and throughout the results reported here, a 3×3 mask matrix (m_m_ = 1) has been used in **Eq.1**.

The second step consists in applying iteratively an uniform threshold over the image and is the most critical step in the segmentation procedure. The user chooses an area in the first image of the time series over which the number of the targets is counted visually and the program starts an iterative procedure that increases gradually the threshold level until the number of targets are retrieved. This procedure can be performed by means of the routine *A_Threshold_Find.m* (see Online Additional Material for a detailed description of its use). The same value of the threshold, T, is applied then to the whole stack of images according to the algorithm:
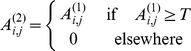
(2)


The third step of the image segmentation consists in the image normalization procedure obtained by applying the mean (**Fig. 1C**) and the maximum filters (**Fig. 1D**) on a (2*m_b_+1*)×(*2m_b_+1*) mask matrix in sequence [13], according to the algorithm:
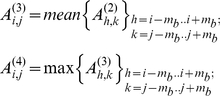
(3)


This procedure allows to discern circular objects on the image. In the case of the SLN particles (**Fig. 1**), we have adopted 3×3 mask matrices (*m_b_* = 1) for these filters: the actual size of these mask matrices must be tailored to the size of the targets of interest.

The final output of this series of filters (see an example in **Fig. 1D** **)** is stored in the matrix *bw3(N,N,M)* by the routine *A_Imagefilters.m* (N is the size of the matrix and M is the number of images in the time stack). The whole set of filters provides a single pixel for each target independent of its size above 3 pixels. The algorithm has also some tolerance on the actual target shape and, being not based on the edge detection, is fairly robust even at high density (small target-target distance) of the targets (**Fig. 1D**). The percentage of accepted targets with respect to the ones visually determined on the first image of a stack, is 90±5% as determined by the analysis of 30 stacks. The extension of the method to variable shapes, for example by setting a constraint on the particle volume, or to a non uniform background, by computing it on sub-matrices of the original frame, can be easily obtained.

### Tracking: non-entangled trajectories

Our strategy is simply to track each of the initially selected particles through the whole set of M images in the time series, 
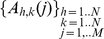
.To this purpose we built (phase B) a series of ADI images in which the differences between one image and its previous one is recorded according to the following algorithm:
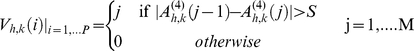
(4)


In Eq. 4, *M* equals the number of images in the time series, P is the number of times at which a motion has been detected in the (h,k) pixel and the threshold value, S, is any positive value in the range [0,1) since we are acting, after the segmentation routine, on binary images. The set 
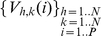
 is a series of P ADI images [13], and is provided by the routine *B_dADI.m* (see the Code S1). The reconstructed stack is updated only when a target moves and the time at which such a motion has occurred (passage time) is then stored in the *V* matrix.

**Figure 2:**
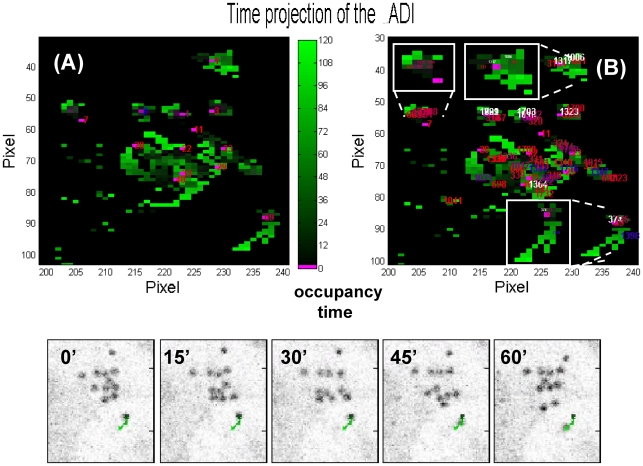
Targets' numbering on the ADI images. Panel A. Result of the superposition of the ADI images with color coding (from dark to light green) for the occupancy time of each pixel. The original time stack (scan1.rar) can be downloaded from the site http://moby.mib.infn.it/~chirico/tracking.html as multi-tiff file. The original image corresponds to the frame ‘0’ reported on the bottom row of the figure. The targets segmented (the threshold parameter in A:image_filters.m is *soglia* = 0.022) on the first image of the stack were tracked through only the first 120 frames of the total 264 of this stack for display purposes. In this case the number of segmented images is 120 (parameter “*numero*” in the code), the parameter *incrementostep* was set to *incrementostep* = 121. The color code indicates the time at which a pixel was occupied in the individual ADI images according to the increasing green level (from dark to light green; the magenta pixels indicate the target position detected on the first image). Only those trajectories longer than 3 frames have been reported (parameters *“lunghezza” and “length_traj”* = 3 in *C_coord_vector.m and D_beta_add_label.m*, respectively). Image B. The same as in Image A but all the targets segmented on every 20 images along the stack (*incrementostep* = 20) have been tracked. The different colors of the target numbers (from red to purple) corresponds to the frames on which the specific target has first been segmented. The white colored text corresponds to those indices that were displaced by at most one pixel since they otherwise superimposed on other indices (see also the Supporting Information S1). The white framed boxes in the image report the zoom of three regions with high density of segmented targets. Notice that the text size is not rescaled while zooming, therefore making difficult to discern the exact position of a target in the original size image (see also the Supporting Information S1; section “Important notes on the color coding”). Bottom row images: frames from the original time stack after 0′, 15′, 30′, 45′ and 60′ superimposed with the reconstructed trajectory (green trace on the gray images) of the target number 69 (panel A). The color bar for the trajectory color coding is displayed between panel A and B and refer to all the images The corresponding movie, **Movie S1**, can be found on the SI and at the site http://moby.mib.infn.it/~chirico/tracking.html.

At this point the algorithm searches (phase C, routine *C_Coord_Vect.m*, in the Code S1) for the connected trajectories of all the segmented targets by working on the ADI matrix *V*. For low density images with non-entangled trajectories of the targets, it is sufficient to follow the motion of all the targets segmented on the first image of the time stack that is taken as reference image. The basic algorithm for this step selects a starting position of the target on the reference image and searches, in its neighborhood (three concentric shells up to three pixels size), the element of the subsequent V matrix whose value corresponds to the lowest j index (see **Eq.4**). Through this procedure, that is repeated until the end of the time stack, the algorithm builds up a 3D matrix in which, for each target, are stored the pixel position and the time value at which that pixel has been occupied by the target. This matrix, called *fine* in the routine *C_Coord_Vect.m* (see Supporting Information S1 for details on this matrix; see also the Code S1 and the section “Important notes on the color coding” in the Supporting Information S1), has the form *fine(traj_length,3,target)*, where *target* is an index that identifies the specific target, *traj_length* is the length in frames of the reconstructed trajectory and in the 3 cells of the second dimension of the matrix are stored the passage time, the x and the y position in the corresponding image of the stack, respectively. The matrix *fine* is particularly useful since it allows the direct parallel inspection of the trajectories on a single 2D image, which is not easily feasible on a movie or its projection, and can also be stored as a list of positions and times at which the selected target has been found for future data analysis. The display of the whole set of retrieved trajectories on a single image is obtained by color coding the passage time memorized in the matrix *fine* (phase D, routine *D_beta_add_label.m*, see Supporting Information S1 and Code S1) as shown for example in **Fig. 2A** (with dark to light green color coding). By selecting the number of the target on this image the user can then also mount a movie of its trajectory superimposed on the original stack of images by means of the routine *G_create_movie.m* (phase G). For example the target number 69 in **Fig. 2A** corresponds to the trajectory reported in the sample frames (from 0′ to 60′, white trace) at the bottom of **Fig. 2** and in the SI (Movie S1).

Regarding the *D_beta_add_label.m* routine it must be noted that the location of the target indices on the image (**Fig. 2**) is made in such a way that the most significant digit of the target index is written at the center of the first pixel of the tracked (sub-)trajectory. However, since MATLAB does not scale the text size while zooming into an image, the user may find convenient to scale down the size of the font size of the targets' indices (*T_F_size* parameter in the *D_beta_add_label.m* routine) and to zoom in the interesting area of the image in order to avoid the superposition of the texts. The text color of the targets' number on the image is coded from red to blue according to the occupancy time of the first pixel of the sub-trajectory ((R,G,B) = 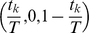, where t_k_ is the occupancy time of the first pixel of the k-th subtrajectory and T is the total stack duration). Finally it is important to note that the targets' indices do not necessarily coincides with the magenta pixels on the images (**Fig. 2A**). In fact these pixels correspond to those targets with trajectories that start in the first frames (see the colormap in Fig. 2 and the examples discussed in the Supporting Information S1 and the movie *S2*).

### Tracking: entangled trajectories

**Figure 3:**
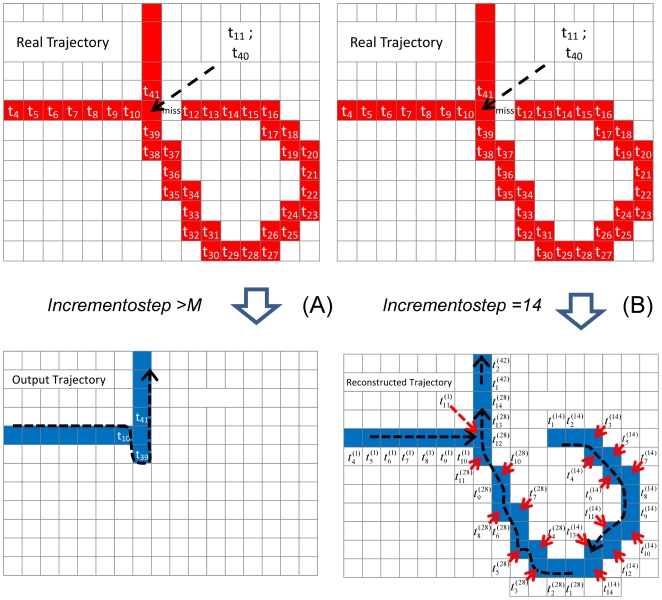
Sketch of the occupancy time for the case of a loop in a single trajectory. One of the positions close to the loop has not been segmented and is missing (indicated as “miss” in the top left panel). In panel A we sketch the situation encountered by the basic tracking algorithm (incrementostep is larger than the total number of frames in the stack, M). In such a case the algorithm takes a detour and skip the loop (bottom panel A). In panel B we report the case incrementostep = 14<M. The tracking algorithm perform a search also starting from the frames 14, 28, … In this case a large fraction of the loop is rebuilt in two distinct sub-trajectories (bottom B panel).

In order to treat dense images and to track entangled trajectories, we apply iteratively the basic tracking algorithm described above by selecting all the targets segmented on every *incrementostep* image along the stack of images and tracing their motion through the remaining part of the time stack. The rationale for repeating the C phase of the algorithm starting from a set of equally spaced images along the time stack, is that an entangled trajectory cannot be fully and continuously retrieved by our as well as by other sequential algorithms due to alternatives found at the branching points that occur on the same (looping) or between different trajectories (crossings). This possibility is sketched in **Fig. 3A**. The basic algorithm follows the nearest occupied pixel with larger (with respect to the current pixel position along the trajectory) occupancy time. In the sketch (**Fig. 3A**) the basic algorithm (incrementostep<M) takes the wrong route simply because close to the crossing one of the positions has not been segmented. This may occur close to the crossing in a loop or at the intersection of crossing trajectories of different targets.

The possibility that we have then exploited is to follow different portions of the same trajectory starting from different frames along the stack. In this way the looping part of a loop can be tracked starting from a position right after the loop (**Fig. 3B**) and reported as a single sub-trajectory. After this procedure, additional specific routines check for the possible crossings of a selected target's trajectory with all the other targets (phase E) and display all the targets whose trajectories cross each other (phase F) on a single image (color codes here for the passage time in the pixel as in phase D) for inspection (see **Fig. 2B**). The evaluation of the crossing between two trajectories is made by checking if the two trajectories belong, at same frame along the stack, to the same hyper-volume, ΔxΔyΔt (routine E_check_crossing.m, see AOM).

**Figure 4:**
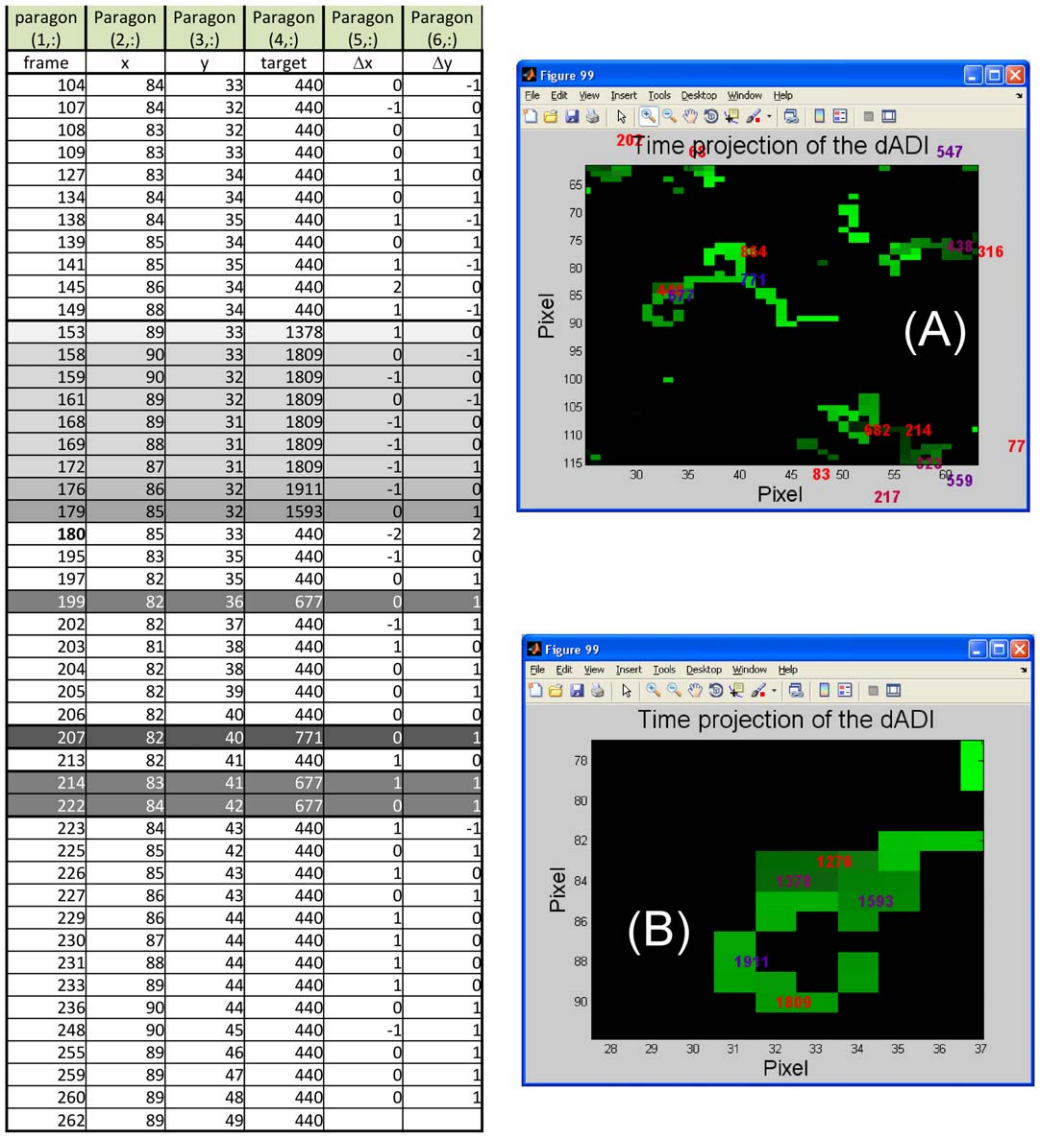
Construction of the trajectory in the case of entanglement. Example of the use of the output (the table *paragon*) of the I phase of the algorithm (*I_Create_Cross_Matrix.m*) for the construction of the trajectory in the case of entanglement. The trajectory taken here as an example presents a loop that can be split in several subtrajectories depending on the *incrementostep* variable (panel A, *incrementostep* = 30 frames; panel B, *incrementostep* = 10 frames). The table reports the final assembling of the trajectory: different colors refer to different targets that are also reported in the panels A and B. In the table the entries are: the frame number, the x and y position in pixels, the target number and the differences between adjacent x and y positions, indicated as Dx and Dy. The corresponding movie of the lymphocyte motion is reported in the SI (the Movie S3).

The final step for the reconstruction of a possible continuous trajectory along the stack is performed then by building a vector (the variable *paragon*) that contains, ordered by the increasing experimental time, all the crossing trajectories (phase I, routine: *I_Create_Cross_Matrix.m*), together with the differences between adjacent positions along the putative trajectory (**Fig. 4**, Δx andΔy in the table). The user have now two choices. She/he inspects the *paragon* vector built during this phase and, by evaluating the continuity of the trajectory (typically one assumes 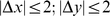), splits the whole vector in subvectors that describe distinct, possibly crossing, trajectories. Alternatively, a specific routine, *K_prepare_paragon.m* (see AOM), performs automatically this test and trajectory splicing according to the threshold value selected by the user (*threshold_paragon* in the *K_prepare_paragon.m* routine), who must then simply validate the splicing and perform possibly the merging of different sub-trajectories in the final complete trajectory, which is then stored in the table paragon and used to mount the trajectory movie (see SI; K_final_movie.m or K_beta_final_movie_traj.m routines in the Code S1 file).

An example is given here for the case of a looping trajectory of a lymphocyte in a lymphonode (**Fig. 4A and B**). In panel A we report the tracking performed with *incrementostep* = 30 frames, in which the loop is assigned to four targets (two of them refer to the loop). The subtrajectories assigned to these targets allow to build a large fraction of the loop (see the rows referring to the targets 440, 677 and 771 in the table in **Fig. 4** (the target 864 offered redundant information). However in order to fully reconstruct the trajectory and in particular the loop, it is necessary to run the C phase of the algorithm with *incrementostep* = 10 frames. In this case five targets are assigned to the loop and by following these subtrajectories it is possible to build the full table of motion reported in the table in **Fig. 4**. The corresponding movie of the lymphocyte motion is reported in the SI (Movie S3).

By tracking the targets from different frames along the stack, the user is then able to reconstruct highly entangled trajectories as it will be further illustrated in the Results section and in the SI by examples.

The user must be aware that, for non entangled trajectories, the same object in the image may correspond to different targets retrieved by the *C_Coord_Vect.m* with *incrementostep<M*. For example the target segmented as number 69 on the image **Fig. 2A** (*incrementostep* = *M+1*), corresponds to four segmented targets (number 69, 374, 725 and 1398) when the stack was analyzed with *incrementostep = 20*. The trajectory of these targets can be followed and built together in order to reconstruct the trajectory reported in the bottom row images of **Fig. 2**.

In the case in which the experiment output is a 3D volume, the above described tracking method can be in principle extended to three dimensions. However it can also be implemented on the 2D projection of the volume despite the decrease in the signal/noise ratio of the projected images.

The algorithm described above produces the path of all circular moving objects selected in the original time series of images even if the target position cannot be segmented in all the frames. This may occur because of large fluctuations of the target emission or because the target temporarily exits from the field of view (in 2 or 3 dimensions). The reconstructed path is visualized by the user on a single output image in which the colors (RGB or, as used here, increasing levels of Green color) code for the time at which the given pixel was occupied (**Figs. 2** **,** **4**). This image can be easily built (routine: *D_beta_add_label.m* in Online Additional Material) by plotting the *V* matrix.

The basic algorithm described in the above section does provide an automatic reconstruction of a large fraction of the trajectories, those not involved in looping or crossing-over with other trajectories. It is worth noticing that this result can be obtained due to the adoption of a segmentation algorithm that is not based on the edge detection and of a tracking algorithm that follows all the segmented targets along the time stack by working on the ADI stack of images and adopts a simple nearest neighbors shell search algorithm. All the information retrieved in this way are then used by the user to build 2D visualization of the trajectories (as in **Figs. 2** **,** **4**). It is also noteworthy that no a priori knowledge or request on the type of motion is made in the algorithm. Entangled or looping trajectories and particle interactions are treated by means of specific routines (phase E, F) that search for the targets that may have interactions (i.e. trajectories that belong to the same hyperspace volume ΔxΔyΔt) and build the corresponding subtrajectories (phase G, H, I) for the subsequent mounting by the user (phase K, see Supporting Information S1).

### Intracellular tracking

**Figure 5:**
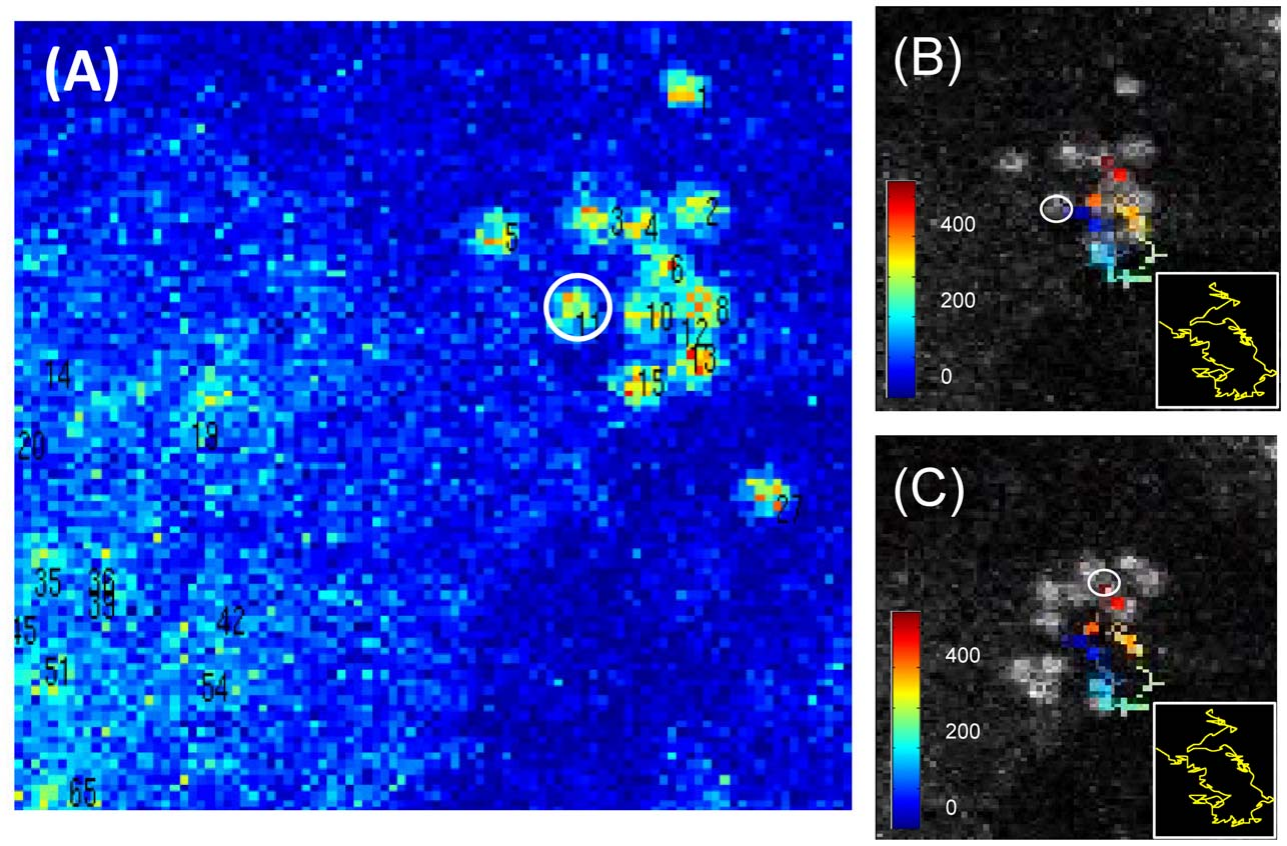
Identification of particles on the image and display of the corresponding trajectory. Panel A reports the initial (frame 1) image of the time stack: the numbers in the image close to each spot are the identification number of the targets that have been segmented (FOV = 22×22 µm^2^). The panels B and C report, superimposed on the first (frame 1, B) and the last (frame 440, C) image of the time series, the trajectory of one of the particle (number 11 in panel A) with a jet colormap that is reported in terms of occupancy time (frames from 1 to 440) in the images. The white framed box report the spline interpolation of the reconstructed trajectory as a thick yellow line. The white circle in the B and C images indicate the initial and the final position, respectively. The corresponding movie is reported, with different color coding of the trajectory, in the Movies S10 and S11.

We have first applied the tracking algorithm to the analysis of time series of images of human lung epithelial cells that internalize solid-lipid nanoparticles labeled with coumarin (c-SLN). The internalization mechanism is not ascertained yet and probably involves the fusion of the SLNs with the membrane phospho-lipids. The coumarin dye carried by the SLNs is found in the cytoplasm only and is characterized by a perinuclear accumulation that has been the subject of further studies. An additional feature of these samples, however, is that beside a relatively large coumarin emission background, distinct bright spots of the size of the microscope point spread function, are detected (**Fig. 1A**). We are interested here in demonstrating the tracking capabilities of our algorithm on the motion of these structures that are likely to be endosomes containing a large number of c-SLNs. They appear to cluster together and to have erratic motions characterized by a wide variability of the displacement (direction and amplitude). The background is relatively high probably due to the release of some coumarin dyes from the c-SLNs once internalized by the cell. We are able to track most (>90%) of the particles initially selected on each time series of images even if the density of the particles in the image is relatively large (≅2 particles/µm^2^ in some regions of interests) as visible from the image reported in **Fig. 5A** and from the movie (Movie S4) reported in the SI. An example of the tracking of one of the segmented particles is given in **Figs. 5B, C** where the trajectory (target 11), in which the occupancy time is color coded (in jet colormap as reported in the panels), is superimposed on the first (**Fig. 5B**) and the last (**Fig. 5C**) image of the time series stack. Although some of the positions along the trajectory are missing, the whole motion can be clearly discerned on the image. A sketch of the whole trajectory, interpolated by spline functions, is reported in the bottom right boxes in panels B and C of **Fig. 5**. as a thick yellow line.

### Trajectory statistical data analysis

**Figure 6:**
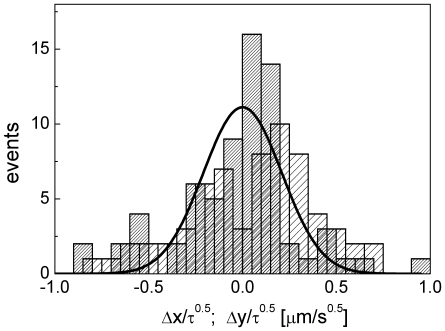
Statistical analysis of the intracellular diffusion of particles. Distribution of the x (sparse pattern) and y (dense pattern) displacements normalized to the square root of the lag time between frames measured on a time series of images of cSLN particles in epithelial cells. Only subsequent detection of the particles have been selected in the analysis. The solid lines are Gaussian functions, 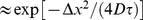, globally fit to both the data sets. The common (globally fitted to the x- and y- displacements) best fit diffusion coefficient of the cSLNs is D = 0.022±0.01 µm^2^/s. We have employed the non linear least square (Levenberg-Marquardt) fitting routine of Origin 7.0 (OriginLab Corp.) with statistical (Poisson) weight on the data.

At short times, the motion of the SLNs appears to be erratic (see the Movies S4 and S5) and it should therefore be analyzed in terms of the Brownian statistics [21]. Actually, one notices that possible interactions with the cellular matrix and/or with other particles seem to induce some directional motion of the particles at long time scales, as visible, for example, in Movie S1. For a Gaussian Markov process the distribution of the displacement (Δx, Δy) measured on the x and y axes in a time lag τ, is Gaussian and is described by the function 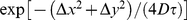, where D is the translational diffusion coefficient of the particle [21]. We are not able to follow continuously the whole trajectory in the experiments and therefore we compute the distribution of the normalized displacements, 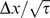 and 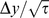 (**Fig. 6**). These normalized displacements are evaluated only for subsequent positions (short time behavior) assumed by each target and averaged over all the targets (≅20) selected in the images. These cases do not always correspond to subsequent frames since in some of the frames we do not detect a change in the particle position. As it can be seen from **Fig. 6**, the histograms of the two normalized displacements can be described by the trial function, 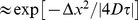, with a global (for the x and y displacements) best fit value D = 0.022±0.003 µm^2^/s, in agreement with those found in most cellular diffusion phenomena [22].

### Cell tracking

It is not our aim to compare the algorithm presented here, tailored for specific applications, with the variety of analysis products existing on the market and available freeware. These have been thoroughly reviewed recently by Hand et al. [12]. We have only taken as a reference the software Volocity (Improvision, Perkin Elmer, UK) [23]. Our choice is motivated only by the wide use of this software in optical microscopy imaging laboratories. The Volocity software does segment a large fraction of the c-SLN particles in the epithelial cells. However, due to the close relative proximity of the SLNs in most of the frames, Volocity is able to provide only very short and fragmented trajectories, that should then be recognized, coupled together and mounted in single longer ones by the user with a lengthy procedure. This situation is mainly the result of the low signal/noise ratio of the analyzed images and the poor edge definition of the targets, that reduces the performance of the edge detection algorithm.

**Figure 7:**
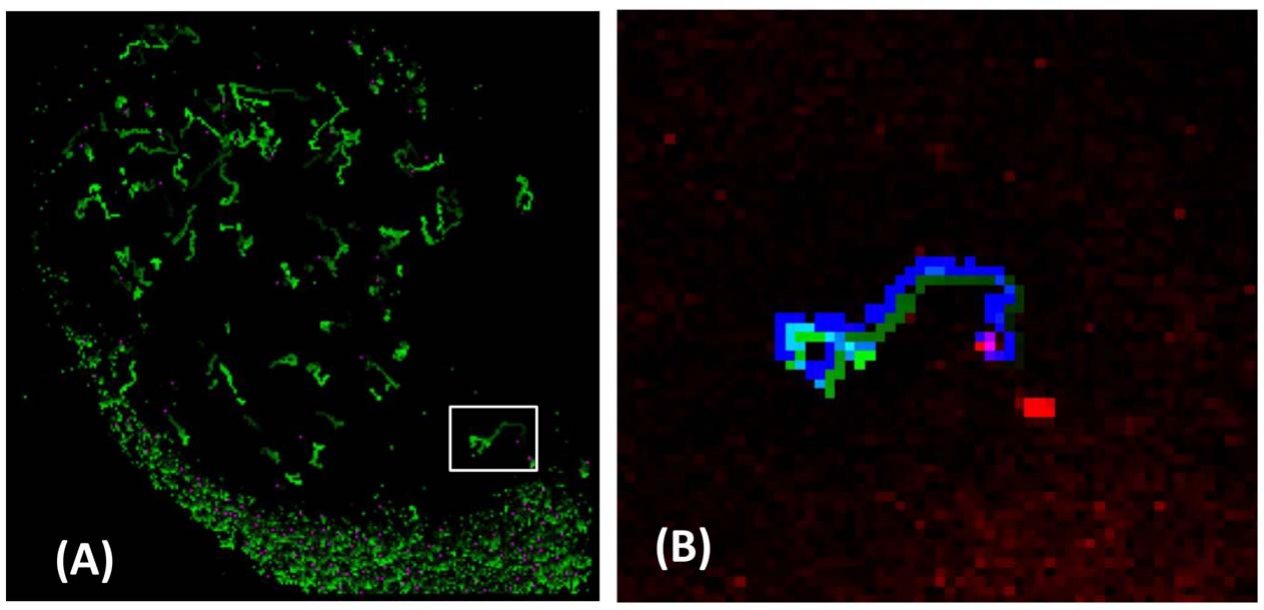
Analysis of lymphocytes trajectories within a ex-vivo lymphonodes kept in physiological conditions. Panels A reports a wide field (256×256 pixels, 2.76 µm pixel size, FOV = 706×706 µm^2^) of the first image of a section of the lymphonodes (LN) together with the trajectories of all the targets that have been segmented. All the images were obtained by z-projection of the experimental volume. The trajectories were recovered by a 2D search on the projected volumes. In the trajectories the experimental time is coded by increasing green levels. Panel B shows a blow-up of the first image of the time series together with the trajectory of one of the visible lymphocytes obtained by the algorithm (the experiment time is coded as increasing green levels and a small shift is applied to the image for display purposes) and of the same lymphocyte as obtained by Volocity (plain blue curve). The resting lymphocytes are coded in red in the image. A white framed square in panel A indicates the position of the trajectory whose blow-up is given in panel B.

On the contrary, Volocity does track efficiently cells on low background images such as in the case of lymphocytes detected by two photon excitation in ex-vivo lymphonodes [19], [20], [24]. This is one of the main topics covered in the UE FP7 research project ENCITE (http://www.encite.org), in which several European groups are involved. The algorithm described here is able to retrieve most (≅80%) of the trajectories of the lymphocytes, as shown in **Fig. 7A**. The only changes that we had to apply to our procedure, with respect to the intracellular tracking of SLNs, was to segment the targets on the 2D projection (sum) of the collected volumes (the experiments provided us with volumes and **Figs. 7** **,** **8** and **9** report their 2D projection), to perform this search on the largest nearest neighbor shell and to enlarge the size of the mean and maximum filter matrices to 11×11 pixels, due to the different optical magnification.

**Figure 8:**
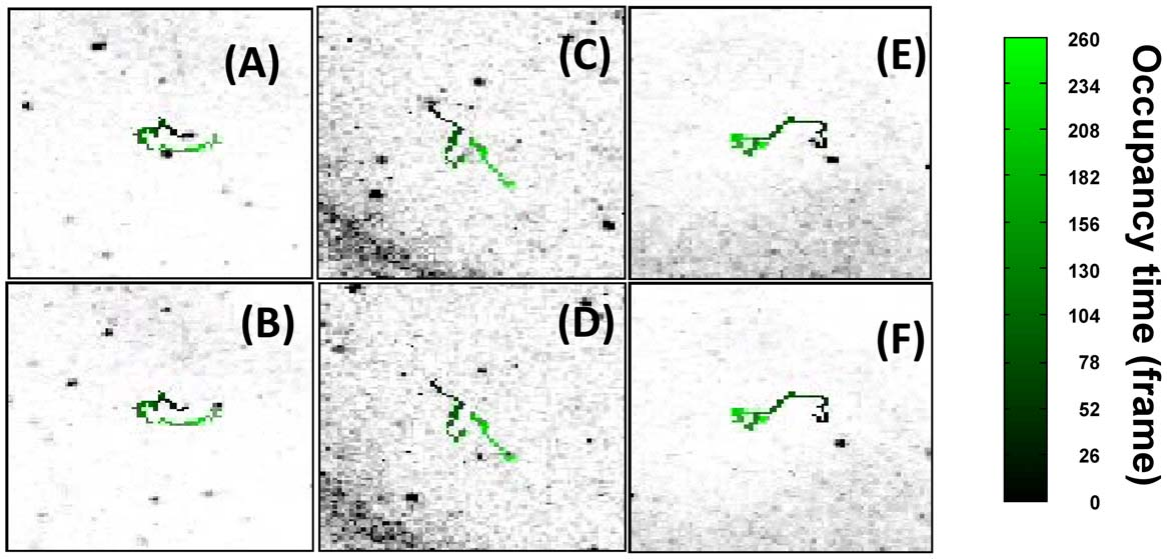
Example of lymphocytes tracking within an ex-vivo lymphonodes imaged under two-photon excitation (λ_exc_ = 800 nm). Panels A to F report the first (A, C and E) and the last (B, D and F) images (FOV = 230×230 µm^2^) of the time series and the corresponding superimposed trajectories in which the experiment time is encoded as green levels as outlined by the reported colormap. These trajectories (reported here in increasing gray levels) have been reconstructed by following the targets segmented only on the first image on the time stack. The LUT of the images have been inverted with respect to **Fig. 7** for display purposes. The movies that correspond to these frames are reported in the SI (Movies S7, S8 and S9).

**Figure 9:**
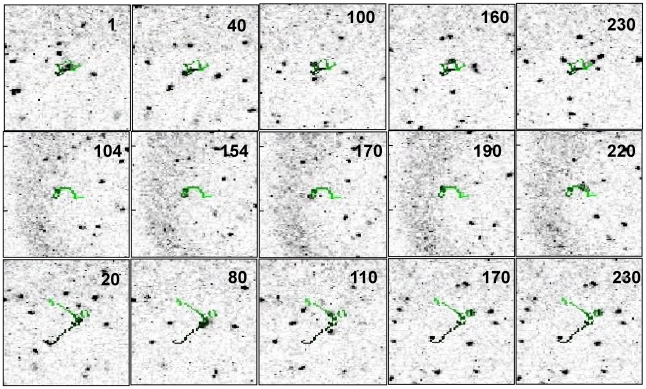
Sample trajectories of lymphocytes within ex-vivo lymphonodes. The images in each of the three rows report a sequence of images from a trajectories of lymphocytes. The field of view of these images is 230×230 µm^2^. The number in each image reports the frame along the time stack. The movies that correspond to these frames are reported in the SI (the Movies S12, S3 and S13). The LUT of the images have been inverted with respect to **Fig. 5B** and **C** and the occupancy time is coded in the trajectory as an increasing green levels (the colormap is the same as in **Fig. 8**).

We have checked that the retrieved trajectories were very similar to the ones obtained by the Volocity analysis (**Fig. 7B**; the corresponding movie is the Movie S6). Therefore the algorithm presented in this report seems to be suited to the lymphocyte motion analysis at least as Volocity does.

When isolated cells with non-entangled trajectories are to be followed, the algorithm can be applied by employing only phases A to G. No need for the repeated segmentation on several equally spaced images along the time stack is needed. These cases are exemplified in **Fig. 8** and in the Movies S7, S8 and S9.

In the case of the most entangled trajectories the segmentation of the targets must be performed on several images along the time stack by selecting a parameter *incrementostep*<*M*. In these cases, the trajectory is built from the combination of the sub-trajectories tracked from different starting images along the stack (phase H, I, K). The procedure has been outlined in the description of the algorithm and exemplified in **Figs. 2** **,** **3** **and** **4**. Only the final inspection of the trajectories superimposed on the image stack, can offer the user the choice to keep the trajectory for subsequent physical analysis or discard it because of the not unique assignment to the cells on the image.

The capability of our algorithm to tackle with entangled and/or crossing trajectories is exemplified by the sequence of images reported in **Fig. 9**, where looping and crossing-over events are visible. It must be stressed that in the reconstruction of a complex trajectory the user is aided by a number of cross-checks implemented in the phases K, I and G of the algorithm, as explained in the Particle Tracking Algorithm section. However, it must be noted that in most of the cases of highly entangled trajectories and cell-cell interactions, is not possible to uniquely assign a target to a specific trajectory and an arbitrary choice must be taken or the trajectory discarded from the analysis. For example, the trajectory reported in the top row of **Fig. 9**, has been obtained by considering four different targets. The trajectories of three of these targets intersect at different times and might be in principle interchanged. In this example (the corresponding movie is found in the Supporting Information as Movie S12), the tracked lymphocyte encounters a second lymphocyte at the frame ≅60 and a third one at the frame ≅76. The user should assign the trajectory and evaluate if interactions are occurring and if the trajectory can be used for further statistical analysis or disregarded due to the not unique assignment.

The middle row of the **Fig. 9** reports the trajectory of a single isolated target in which a tight loop is recognized and followed to large extent by the algorithm. In the bottom row of **Fig. 9** two closely spaced loops are reconstructed in the target trajectory. From the corresponding movies reported in the Supporting Information (Movie S13 for the bottom row and Movie S3 for the middle row of **Fig. 9**), it can be seen that the missing stretches in the trajectory (particularly in the bottom row of **Fig. 9**) correspond indeed to frames in which the target is probably exiting from the observation planes.

The algorithm presented here has a number of additional useful features when compared to the chosen reference software, Volocity, if applied to the lymphocyte motion analysis. In the case of the images of lymphocytes within an ex-vivo lymphonodes, the background is much less evident than that found for the c-SLNs images, even after performing the 2D projection, and the most difficult and immunologically relevant issue is the possibility to detect contacts between lymphocytes of different types. During the immunological response to an external stimulus these interactions are triggered by cell-cell interactions and the resulting lymphocyte trajectories can be entangled or crossing each other. The cases in which two trajectories are intersecting are the most difficult to be treated, but often the more relevant. Intersection can also occur because we are measuring on a 3D volume and two trajectories happen to have the same (x,y) coordinate, though at different heights (z coordinate) in the volume. However the limited z resolution of optical (even confocal or non linear excitation) microscopes often induces cross-talk between adjacent z planes. In other cases, the trajectories are touching because two lymphocytes of the same type (and staining) pass by each other by chance or because two lymphocytes of different types (and staining) are recovered on the same image channel because of cross-talk between the image channels.

The algorithm presented here has the capability to segment most of the cases related to cell interactions, and therefore produces long trajectories approximately lasting one third of the whole duration of the experimental observation (60′–90′). In this crucial step the user is aided by specific routines that single out the targets putatively involved in crossing-over and interactions and evaluate the continuity of the corresponding sub-trajectories as outlined in **Fig. 4**. The Volocity software, on the contrary, reports entangled and touching trajectories as a collection of fragmented and uncorrelated sub-trajectories that must be carefully inspected by the user and possibly mounted in longer ones with lengthy procedures.

It was not our aim to develop a fully automatic and general purpose tracking algorithm and therefore we do not extend further the comparison of our algorithm with other software packages. A comparison to the recently published work by the Degerman group [7] is however particularly useful. The algorithm presented in [7] is devoted to the segmentation and tracking of neural stem/progenitor cells in vivo and it is based on a highly sophisticated iterative method. In this biological system the cells can fuse and change in shape and the analysis of the motion can be extremely complex. Degerman and coworkers [7] extend the algorithm previously developed by the same group, starting from the Chan and Vese algorithm [15], by taking into account also the growing and pruning of the neural cells. This is a case in which a number of assumptions on the type of the cell dynamics (both for the cell shape and for the cell overall displacement) is needed and taken into account by solving the propagator equation for a smooth function [7], [15]. In order to track the cell motion (and their shape change) Degerman et al. [7] make use of a stochastic model (hidden Markov model) in order to estimate the probability that certain segmented targets belong to a specific track. This type of approach is therefore complementary to ours in several point. First, regarding the size of the cells that are, in our case, more or less small (3–10 pixels) circular (or spherical in 3D) closed objects whose shape cannot and should not be solved accurately from the image, and whose volume is not dramatically evolving in time. Second, the tracking is performed in our case at a low level, by making no assumption on the type of motion and by following all the segmented objects through the time series of images or volumes. It is at the basis of our approach the assumption that the user will then need to validate the reconstituted trajectories and to apply models to analyze the target motion. Third, in the present case the segmentation can be performed in almost any freeware platform and the tracking algorithm needs a minimum of coding (subtraction of subsequent normalized matrices).

### Conclusions

We have described a simple algorithm for the segmentation and the tracking of particles on time series of optical microscopy images based only on a set of size sensitive filters and with no a priori requirement on the exact shape of the objects to be segmented and their type of motion. The procedure, implemented here on the MATLAB (7.7) platform can be easily extended to faster performing environments, such as ImageJ (http://rsbweb.nih.gov/ij/) or CVI (NI, http://www.ni.com), and has been tested on nanoparticles diffusing through cell cytoplasm and on lymphotcytes diffusing through the lymphonode tissue. The segmentation part of the algorithm can be further tailored by changing or skipping some of the applied filters while the tracking part of the algorithm, based on the application of the ADI technology [13], allows to track the whole set of particles initially selected by the user and to visualize all the corresponding trajectories on a single 2D image.

In summary, the tests presented indicate that the method adopted here to segment the targets on the images and to encode time in the time series of images for a specific target, allows to treat efficiently the most critical issues in the analysis of cell motion in in-vivo studies, namely cell interaction and entangled trajectories, even on low signal/noise images and provides the user with an efficient visualization of the trajectories for further analysis.

## Supporting Information

Supporting Information S1 This file provides all the Supporting Information. The two main sections are devoted to (1) a detailed description of all the routines and of the linked use for segmentation and tracking and (2) to some considerations on the problems related to the position of the target numbers on the images.
(0.62 MB PDF)

Code S1 This is an archive that contains all the matlab routines (some of them in revised beta version) needed to analyze the time stack of images. The sample stacks and this same zip archive can be downloaded from http://moby.mib.infn.it/~chirico/tracking.html.
(0.02 MB ZIP)

Movie S1 This is a movie that reports the kinetics of SLNs nanoparticles in cells. The corresponding image is a blow up (15×15 µm^2^) of Movie S4. The trajectory of the target is reported in all the frames as a green track. The green colors of the trajectory map the occupancy time as reported by the colorbar of Fig. 2.
(5.91 MB AVI)

Movie S2 This is an animated GIF file that visualize the effect, on the positioning of the number of the specific target, of zooming into an image with MATLAB.
(0.60 MB GIF)

Movie S3 This movie shows the motion of a lymphocyte close to the external capsule of the lymphonodes. The cell appears (coming from above or below planes) in the image approximately at frame 99–101 and it is first segmented on frame 102 and tracked from this frame on. An interaction between this cell and a cell coming from the upper part of the image occurs between the frames 204 and 226, approximately. We cannot be sure that the trajectory built from frames 226 on is the one that refers to the cell initially segmented on the image at frame 102. The cell, in the frame 130–180, performs a loop and inverts its motion. The trajectory green colors code for the occupancy time as in Fig. 2.
(12.99 MB AVI)

Movie S4 The movie shows the kinetics of SLNs in cells on a wide FOV (120×120 µm^2^).
(89.08 MB AVI)

Movie S5 The movie shows the kinetics of SLNs in cells on a wide FOV (60×60 µm^2^).
(57.35 MB AVI)

Movie S6 Movie reporting the motion of a lymphocyte within a lymphonodes. Comparison between the trajectory retrieved by the Volocity (green) software and our algorithm (blue). The mobile lymphocyte is marked in red. A second immobile lymphocyte is present in the movie.
(0.88 MB AVI)

Movie S7 The target starts from the upper right end of the trajectory. A second target happens to lie on the same trajectory in the very first frames of the stack (on the bottom part of the trajectory). The target assigned to the trajectory moves first to the left performing two small loops at frame ≅ 80 and ≅ 100. The it closes up turning to the right. The color map of the image is an inverted gray colormap. The trajectory is reported in green levels with the same colormap as in Figs. 8, 9.
(12.99 MB AVI)

Movie S8 The trajectory illustrates the motion of a lymphocyte in which a large loop is present. There is no direct interaction with other cells and no ambiguity in the trajectory assignment. The color map of the image is an inverted gray colormap. The trajectory is reported in green levels with the same colormap as in Figs. 8, 9.
(12.99 MB AVI)

Movie S9 The trajectory reports the case of an isolated loop. The target starts the loop at frames ≅ 130. Between frames 150 and 180 the target has not been sesgmented, probably because it exited the imaged volume. The color map of the image is an inverted gray colormap. The trajectory is reported in green levels with the same colormap as in Figs. 8, 9.
(12.99 MB AVI)

Movie S10 The movies report the reconstruction of the trajectory of one SLN in the cells (target 11 in Fig. 4). The images are reported in direct gray levels (all the other movies are in inverted gray levels). The trajectory lasts for 440 frames and therefore two different color rendering is given here. In this movie one every two frames are inserted in the movie and the whole trajectory is rendered in jet(128) colormap on all the frames of the movie. It is noteworthy that, despite the high density of targets in the image and the several interactions between them (20<frames<110, 180<frame<210; 320<frame<440) the trajectory is fully reconstructed with a low level of ambiguity.
(21.39 MB AVI)

Movie S11 The movie reports the reconstruction of the trajectory of one SLN in the cells (target 11 in Fig. 4). The images are reported in direct gray levels (all the other movies are in inverted gray levels). The trajectory lasts for 440 frames and therefore two different color rendering is given here. In this movie all the frames are inserted and the trajectory is reproduced in two different parts, both rendered in the jet(128) colormap. For the first 220 frames only the first half of the trajectory is reported on the movie. For the second 220 frames only the second half of the trajectory is reported on the movie, in the same jet(128) colormap. It is noteworthy that, despite the high density of targets in the image and the several interactions between them (20<frames<110, 180<frame<210; 320<frame<440) the trajectory is fully reconstructed with a low level of ambiguity.
(21.69 MB AVI)

Movie S12 The movie represents one of the most entangled trajectory found in the original image of lymphocyte in a mouse lymphonodes. The choice of the connection between different sub-trajectories is only indicative of the potential application of the software. The cell is segmented on the very first image of the stack. Up to the frame 60 there is no ambiguity on the assignment of the trajectory. At this frame the original target meets a second target coming from the third quadrant of the image. At the frame 73 these two targets encounter a third target coming from the upper part of the image. From frame 80 to 190 the trajectory shows no ambiguity in the assignment. At frame 190 a second lymphocyte appears in the image and splits from the original one. This is probably due to an interaction between lymphocytes on different z planes. This trajectory is reported here only with the purpose of showing the possibility of reconstruction of complex trajectories. In the analysis of the cell interactions such a trajectory should be discarded due to the large ambiguity in the assignment of the cells. The color map of the image is an inverted gray colormap. The trajectory is reported in green levels with the same colormap as in Figs. 8, 9.
(12.99 MB AVI)

Movie S13 This movie reports the trajectory of a cell that interacts partially (frames 80–180) with the cell reported in Movie S12. Also in this case there is some level of ambiguity in the assignment of the trajectory that must be settled by the user or the trajectory should be discarded from the analysis. The motion of the cell presents two adjacent loops (frames 70–90 and frames 150–200) and an inversion of the motion (frame 105). The color map of the image is an inverted gray colormap. The trajectory is reported in green levels with the same colormap as in Figs. 8, 9.
(12.99 MB AVI)
