# Supplementary material for: Accumulative Difference Image Protocol for Particle Tracking in Fluorescence Microscopy Tested in Mouse Lymphonodes
Source: PLoS One. 2010 Aug 17;5(8):e12216. doi: 10.1371/journal.pone.0012216 (PMC2923183; doi:10.1371/journal.pone.0012216)
Supplement: Supporting Information S1 — This file provides all the Supporting Information. The two main sections are devoted to (1) a detailed description of all the routines and of the linked use for segmentation and tracking and (2) to some considerations on the problems related to the position of the target numbers on the images. (0.62 MB PDF) [file pone.0012216.s001.pdf]

SUPPORTING INFORMATION

Accumulative Difference Image Protocol for Particle Tracking in Fluorescence Microscopy tested in Mouse Lymphonodes.

Carlo E. Villa[1], Michele Caccia[1], Laura Sironi[1], Laura D'Alfonso[1], Maddalena Collini[1], Ilaria Rivolta[2], Giuseppe Miserocchi[2], Tatiana Gorletta[3], Ivan Zanoni[3], Francesca Granucci[3] and Giuseppe Chirico[1,],*
[1]Dipartimento di Fisica, Università degli Studi di Milano Bicocca, Milano, Italy.
[2]Dipartimento di Medicina Sperimentale, Università degli Studi di Milano Bicocca, Monza, Italy.
[3]Dipartimento di Biotecnologie e Bioscienze, Università degli Studi di Milano Bicocca, Milano, Italy.

Description of the code and instruction for its use.

The routines have been tested on MATLAB R2009a (Mathworks Inc.) with Image Processing Toolbox.

The algorithm is implemented in ten steps or phases (from A to K) that are coded in different matlab files. Several tools are provided in the form of additional routines, to provide estimates of the threshold on the images, to test the output and to help the user in selecting the targets and to reconstruct trajectories in the most critical cases in which some trajectories are intersecting each other. The main routines are devoted to the settings of the filters for the images, the compilation of the ADI image, the visualization of the output and the test on the final output. At the end of each step, the user can check for the output and feed it to the next routine. The routines, that are described hereafter, are set for a size 256x256 of the images.

In all the routines the input parameters are set in the first lines of the routine. The end of this input section is marked by the comment line:*"%%%%%% END OF INPUT"*. Throughout this document the variables are written in `Courier New 11`, and the name of the routines are in *Italic Calibri 11*.

All the routines are available as Online Additional Material in the code_beta.rar file.

PHASE A: FILTERS' SETTINGS

A_IMAGEFILTERS_BETA.M

The first routine applies the filters to the images in the stack. The routine uses the routine *rd_img16_gib_gamma.m* to read in the series of tiff images.

Input:

- The path and the name of the original images is stored in the matrix "`dafiltrare`". The name of the stack must be inserted in the first line. For example if one wants to read the stack of images "scan1.tif" the first line of the routines reads:

```
dafiltrare = rd_img16_gib('scan1.tif');
```

- The number of the last frame to be read from the stack is stored in the variable `numero`.
- The number of the first frame to be read from the stack is stored in the variable `init_frame`.
- The frame rate with which the reading of the stack is performed is stored in the variable `fr`. Example: if `fr = 2,` one every two frames are read in. Please notice that the variable numero is redefined in this routine, and so used in the subsequent routines, as:

```
numero = (numero - init_frame)/fr + 1;
```

Therefore the variable `numero` contains, in the rest of the code, the total number of frames to be analyzed.

- The threshold value is stored in the variable `soglia`. After a first trial with this routine, the value of the threshold can and should be refined by means of the routine <u>*A_test_thresholds.m.*</u>
- The number of pixels that defines the outer edge of the image that is excluded from the analysis is stored in the variable `frame_edge.` The default value is `frame_edge` =5.
- The matrix dimension for the mean and maximum filters is stored in the variable `larghezzaintornomedia`: this value must be odd and greater than 1.

Output:

There is no specific output on the screen. The main internal output is the matrix `bw3(N,M,K)`, that stores the binary stack of images. N and M are as default set `N=M=256`. The index K is equal to the input number of images to be analyzed, `K=numero`.

<u>Related routines (phase A).</u>

<u>*A_test_thresholds.m.*</u> This routine is designed to help the user to estimate of the threshold value by applying iteratively the main algorithm, as used in *A_binarizzaprova.m,* to the first "`numero_t`" images of the stack. Notice that this routine must be run after running for the first time *A_binarizzaprova.m*, because the stack of images is read into the memory by this routine. The routine computes the average number of targets segmented in the first "`numero_t`" images of the stack with an increasing value of the threshold from "`soglia`" to "`soglia + num_iter*incrementosoglia`". This computation is can be limited to a Region of Interest (ROI) delimited by rectangle with vertices, (x0,y0)-(x1,y1).

Input

- `numero_t`: number of images on which the number of targets segmented (default is 5) is averaged.
- `(x0,y0) – (x1,y1)`: coordinates of the box on which the computation of the average number of segmented targets is done (default is (30,120) – (80,170));
- `num_iter`: number of values of the threshold on which (default is 20);
- `soglia`: is the starting value of the threshold;
- `incrementosoglia`: increment of the variable `soglia`.

While running, the routine writes on the prompt screen the actual value of the threshold used. The user can visually choose which one is the right choice. In the results reported in the Ms. we usually preferred to visually count the target in a specific area of interest and check the identified target.

Output: the routine stores in the matrix `bw4(256,256,numero,num_iter)` the segmented images. The i-th segmented image obtained with the threshold value, `soglia + k*incrementosoglia`, is stored in the matrix `bw4(:,:,i,k)`. This routine opens two figures that reports the first image (colormap=jet) to be segmented and the corresponding image segmentation obtained with the value of the threshold = `soglia + incrementosoglia*num_iter/2` (colormap=hot).
The estimate of the threshold can also be gained from the plot of the vector testsoglia(num_iter,2). This vector is plotted in the figure 5 at the end of the run.

- `testsoglia(num_iter,2)`: contains the average number of segmented targets on the first numero images of the stack `(testsoglia(:,2))` as a function of the threshold `(testsoglia(:,1))`.
- `Bw4(:,:,i,k)`: segmented images. The i-th segmented image obtained with the threshold value, `soglia + k*incrementosoglia`, is stored in the matrix `bw4(:,:,i,k)`.
- Figures.
  Fig.1. Original first image (colormap jet).
  Fig.2. First segmented image with threshold=soglia.
  Fig.3 First segmented image with threshold=soglia + num_iter*incrementosoglia/2.
  Fig.4 First segmented image with threshold=soglia + num_iter*incrementosoglia.
  Fig.5. Plot of the average number of the segmented targets as a function of the threshold.

A_draw_binaries.m. To test the output of *A_test_thresholds.m* the user runs this routine which provides a series of figures related to the last image segmented in the series, that is the image number=`numero` of

the stack. The variable numero, that assign the total number of images to be read and analyzed, is set by the user in the first lines of the *A_imagefilters.m*.

- Fig.1: Image after the Minimum filter application.
- Fig.2: Image after the threshold filter
- Fig.3: figure after the Mean filter application
- Fig.4: Image after the maximum filter application
- Fig.5: original image

PHASE B: COMPILE ADI

B_DADI.M

This routine builds the ADI from the time stack.

Input.

g: total number of images of which the ADI matrix must be built. The default value is g = numero. It can be changed in the first lines of the routine.

Output:

vel(256,256,g): this is the ADI matrix for the segmented targets and is built from the binary image stack bw3.

PHASE C: COMPILE THE TRAJECTORIES.

C_COORD_VECTOR.M

This routines actually creates the matrix in which the trajectories of all the segmented targets are stored. These are written as (x,y) coordinates and the corresponding passage time, for each target. The targets that are followed in time are those which are segmented every incrementostep images in the original time stack. This option is essential for the pathological cases of loops or intersecting or interwound trajectories. These cases are then built by composing sub-trajectories detected from different starting images chosen every incrementostep.

During this routine the matrix vel is duplicated into vel2, since the vel matrix is destroyed during the tracking procedure.

Input.

`lunghezza`: this parameter sets a threshold to the minimum length in pixel of the trajectories that are stored. The default value is 20.

`incrementostep`: The targets which are followed in time are those which are segmented every `incrementostep` images in the original time stack. If `incrementostep >= numero`, the tracking algorithm is performed only once through the whole stack. If `incrementostep < numero`, the tracking algorithm is performed (`numero/incrementostep -1`) times, starting from one every `incrementostep` frames through the whole stack.

Output.

The main output of these routines is the matrix "`fine`". This is the matrix where the software stores all the data that define the trajectories of all the identified targets. The number of segmented targets is stored in the variable `contatore1`, that is also the screen output of the routine. The `fine` matrix is stored for the i-th target (`1 ≤ i ≤ contatore1`) as:

- `fine(:,1,i)`: passage times on the (x,y) pixel.
- `fine(:,2,i)`: X coordinate of the pixel at which the target was found at this passage time.
- `fine(:,3,i)`: Y coordinate of the pixel at which the target was found at this passage time.

The number of items in the first dimension of the matrix `fine` depends on the target (and therefore on the index i), since it is the length of its trajectory.

NOTE: It is important to notice that not all the targets found by this routine corresponds to really different targets in the image, since all the targets that are found every `incrementostep` images are followed in the ADI image. The variable `contatore1` stores the number of all the targets that have been segmented on all the `numero/incrementostep` images, irrespective of the control on the length of the trajectory. This can be a large number. However the size of the third component of the matrix fine is the value `contatore`, that is typically much smaller than `contatore1`, due to the constraint imposed on the minimum length of the trajectory (that can be relaxed by the user acting on the variable `lunghezza`).

Related routines (phase C).

C_Test_Visualize_Vel.m. This routine provides the user with a fast parallel picture of the trajectories of all the targets that have been segmented, whose number is stored in the variable `contatore`. This image is opened in the window entitled "Parallel Visualization of the trajectories". The color codes, in increasing levels of green, the passage time of the target in the pixels, normalized to the length of the trajectory for

each target. The magenta point on each trajectory indicates the starting pixel of a trajectory that origins from the very first image of the stack. Two figures are opened with a different choice of background for better evaluating the whole development of the trajectory. In this figures no number of targets is reported (see phase D for target number encoding).

C_Test_length.m. The length of each target's trajectory (in pixels) is provided to the user by the routine *lunghezzaFINE.m*. that can be run after having the compilation of the matrix `fine`. The length of the trajectory for the i-th target is stored, after the run of lunghezzaFINE.m, in the variable `contatorepunti(i)`.

Input:

`max_duration`: this is the maximum duration of the trajectory that is tested. This value can be equal to the total number of frames (`numero`).

Output:

The figure reporting the plot of the trajectory duration as a function of the number of the target.

C_ee_FINE.m. This routine computes for each target the end-to-end distance of the trajectory and stores it in the vector `contatorepunti2`. This is the most valuable parameter for detecting a putative loop in the trajectory. Those trajectories to which corresponds a small value of the end-to-end distance, are likely to be entangled.

Output:

The routine provides a plot of the end-to-end distance as a function of the index of the trajectory/target.

PHASE D: VISUALIZE THE SINGLE TRAJECTORY

D_BETA_ADD_LABEL.M

D_add_label.m. This routine creates a display of the trajectories (the matrix `fine`) together with the number of the segmented target superimposed on the image itself. The color codes (from dark to light) the passage time on the specific pixel of the target whose index number is written on the image close to the position of the first appearance of the target in the time stack (i.e. the frame from which the target has been followed along the stack). It opens a new figure window entitled "Time projection of the dADI". The total number of the displayed target numbers can be limited, with respect to the total `contatore` value, by setting a minimum value of the trajectory length to be displayed.

Input:

`lenght_traj`: this is the minimum value of the length of the trajectory for which the corresponding target number is displayed on the figure.

`T_F_size`: the size of the target numbers marked on the image, default is 10. The user should resize the image in order to read correctly the target numbers in highly dense images.

`D_T_F_size`: the increment of the size of the target numbers marked on the image. The default value is 0. Additional to this feature, the user should resize the image in order to read correctly the target numbers in highly dense images. The algorithm for the text sizing is:

```
colore=max(fine(1,1,:)); % colore = maximum occupancy time in the trajectory

T_F_size = T_F_size_0 + D_T_F_size*fine(1,1,i)/colore;
                          %% fine(1,1,i) represents the i-th occupancy time.
```

Output:

the figure that reports the `vel` (or ADI) matrix superimposed on the first binarized image of the stack and with the number of the corresponding target marked on the image itself. The color of the target is chosen between red and blue (RGB) according to the image from which the tracking has started. The algorithm for the color coding of the number of the target on the image is:

```
ColorRGB = [ 1-(fine(1,1,i)/colore); 0; (fine(1,1,i)/colore)]
```

Therefore the most red numbers on the images corresponds to targets that have been segmented on the first image of the stack and the most blue ones corresponds to those segmented on the last image in the stack from which a tracking has been performed. The ADI image is coded in green levels (passage time from dark to light green).

The routine performs also a search for possible superposition of text on the image. In the case that two indices corresponds to the same pixel position (this may occur for `incrementostep<numero`), the routine displaces the largest occupancy time in the closest nearest neighbors and changes the color of the displaced text to white.

Please notice that the text of the index is written by MATLAB in such a way that the most significant digit lies on the assigned pixel. However, for highly dense images, the user should zoom the image in order to see correctly placed the indices. See also *Regarding_D_add_label.pdf* and *AnimatedZoom_forTextPositioning.gif* in the OAM, to this regard.

D_DRAW_TARGET_RMS.M

This routine provides the user with a visualization of the trajectory of the selected target. It is suggested that the user run "D_add_label.m" before this step in order to identify the most interesting targets. The input parameter to be set in the routine is the order number of the selected target (variable: punto) and the output is a series of images and plots.

Input:

- punto: is the variable in which to store the number of the target of which one wants to display the trajectory.
- High_T: is the value of the threshold to be used to display the images in Fig.3 (see output below. This value enhance the details of the images and is used only for display purposes.

Output:

- Fig.1: this figure, whose title is "TRACE" displays the trajectory of the selected target (specified by the variable punto) in jet colormap. The color codes for the increasing passage time along the trajectory, normalized to the length of the trajectory for the specified target.
- Fig.2. this figure, whose title is "rms displacement" ($\left\langle \left| x(\tau) - x(0) \right|^2 \right\rangle$) displays the root mean square displacement of the selected target.
- Fig.3. this figure displays the trajectory of the selected target superimposed on the first and the last image. The trajectory is displayed with the occupancy time in increasing levels of blue color.

PHASE E: ANALYZE TRACKS FOR CROSSINGS

E_CHECK_CROSSINGS.M

This routine has been devised to detect the entanglement points in trajectories. The user must select a target and the routine analyzes its trajectory looking for space-time points (x,y,t) of other targets' trajectories that lie "close" to the selected trajectory. This test is performed by selecting a (3D) volume whose size, $\Delta x \Delta y \Delta t$, is set by the user in the routine. The output is a table of targets whose trajectories lies within the preset xyt volume with the trajectory of the target chosen to be analyzed.

Input.

Punto_iniziale: this variable contains the number of the target whose trajectory must be scanned for possible interactions with other targets.

delta_t: this is the half of the time axis of the hypersphere. The default value is 3.

`delta_x`: this is the half of the x-axis of the hypersphere. The default value is 2.

`delta_y`: this is the half of the y-axis of the hypersphere. The default value is 2.

The routine searches for the instances that corresponds to trajectories that, at times t_1 and t_2 that lies within `2×delta_t +1`, passes for points (x_1,y_1) and (x_2,y_2) in the image that lie within a cube of size `(2×delta_x+1)×( 2×delta_y+1)`. The number of couples of such points (not trajectories but points along the trajectories) is stored in the variable `controllo_contatore`.

Output.

The output of the routine is the matrix `controllo_tabella` whose size is `controllo_contatore` ×8. The format is the following.

- `controllo_tabella(i,1)` = index of the trajectory that runs close to the trajectory whose index is stored in `controllo_tabella(i,5)`. The passage times, t_1, and the positions (x_1,y_1) that have satisfied the space-time distance condition with a second point characterized by the coordinates (t_2,x_2,y_2), are stored in `controllo_tabella(i,2-4)=` (t_1,x_1,y_1) and `controllo_tabella(i,6-8)=` (t_2,x_2,y_2).

  An example of the `controllo_tabella` is given hereafter.

Targ0	X0	Y0	T0	Targ1	X1	Y1	T1
290	113	226	117	594	113	226	117
290	114	226	122	594	114	226	122
290	117	229	122	594	117	229	122
290	117	229	122	604	117	229	122
290	117	229	122	612	117	229	122
290	117	229	122	615	117	229	122
290	113	226	117	921	113	226	117
290	114	226	122	921	114	226	122
290	117	229	122	921	117	229	122
290	113	226	117	923	113	226	117
290	114	226	122	923	114	226	122
290	117	229	122	923	117	229	122
290	114	226	122	940	115	226	122

Targ0 is the analyzed target: Targ0=Punto_iniziale. Targ1 is the index of the target that has interactions with Punto_iniziale. (X0,Y0,T0) and (X1,Y1,T1)) are the space-time points of the trajectory that satisfies the interaction criterion:

$$X1 - X0 < \Delta x \qquad \text{AND} \qquad Y1 - Y0 < \Delta y \qquad \text{AND} \qquad T1 - T0 < \Delta T$$

Therefore in the above example, the targets number 594, 604, 612, 615, 921, 923 and 940 may have interactions with the target 290.

PHASE F: DISPLAY TRACKS WITH CROSSINGS

F_CROSS_ADD_LABEL.M

This routine displays the trajectories of those targets that present some interactions with the target whose index is `Punto_iniziale`, as selected in phase E.

Input.

`PuntoIniziale`: this value is the index of the trajectory/target of which we would like to consider possible superpositions with other targets' trajectories. This input is set in the E_check_crossings.m.

Output.

A figure is opened reporting the ADI image on which the indices of the trajectories that may have some superposition with the chosen target's trajectory, as set by the content of the matrix controllo_tabella, are displayed. If the value of `PuntoIniziale` does not correspond to a target with interactions with other targets only the trajectory of the target `PuntoIniziale` will be shown on the image.

PHASE G: MOUNTING OF THE MOVIE IN ABSENCE OF CROSSINGS

G_CREATE_MOVIE.M

This routine creates a movie of the trajectory of the selected target and allows to perform simple movie operations, such as zoom, back and forward, pause and frame-by-frame visualization of all the trajectories that are linked to the selected target by the matrix `controllo_tabella`. This routine must then be run after E_check_crossings.m (that creates the `controllo_tabella` table) and after setting the proper colormap.

Each of the targets present in the table controllo_tabella, and therefore possibly related to the target with index Punto_iniziale, is reported in the images of the stack as a moving circle with colors that falls within the colormap cmap2. All the circles are reported on the images: they start moving when the corresponding target moves on the image and disappear when the target has been lost on the image. The image is displayed instead as a 8 bits color map according to the colormap cmap1. The algorithm for the selection of the maps reads:

```
cmap  = colormap(jet(128)); %% this for the image, default jet
cmap2 = colormap(hsv(128)); %% this for the circles that identify the targets
cmap1(1:128,:)=cmap;
cmap1(129:256,:)=cmap2;
cmap1(256,:)=[1 1 1]; %% this is for the saturated pixels: white.
```

Input:

High_T: this is the threshold value used to amplify the contrast on the image.

a: is the actual number of images to be mounted in the movie. The default is a=numero, i.e. all the numero images are mounted.

Max_tab: is the maximum number of targets linked to the target analyzed (whose index is Punto_iniziale) to be displayed on the movie. This number should be larger than the rows in the table controllo_tabella. The default value is Max_tab=30, which more than sufficient to treat all the linked targets. It can be lowered by the user if only some of the linked targets are to be followed on the movie.

cmap and cmap2. These are the color maps for the image and for the circles that describe the position of the targets. The default values are: jet(128) for the image and hsv(128) for the circles. This parameter can be changed by acting on the input parameter lut_flag in the following way:

```
lut_flag = 1; % lut_flag = 0 --> jet, lut_flag = 1 --> hot
```

Output:

The movie is mounted by copying the frames initially displayed on the screen on an avi file, whose name is created as 'Gmovie.avi'.

The Movie is also opened on the screen by means of the Matlab encoded avi player, implay.

PHASE H: DISPLAY CONTIGUOUS TRAJECTORIES

H_TRACK_3D.M

This routine allows the user to superimpose the trajectories that refer to the targets that have been found to have interactions with the analyzed target (whose index is `Punto_iniziale`). It displays a 3D plot in which the trajectory found for each of the targets listed in the table `controllo_tabella`, are displayed on different z planes. The z coordinate corresponds to the order with which the target appears in the `controllo_tabella`. The plot is opened first on the z projection: all the trajectories are superimposed. The user can then use the “rotate 3D” button on the image window menu to visualize different projections.

The starting point of each trajectory is marked by a red sphere. The actual target number that corresponds to a trajectory reported in this plot can be gained by the inspection of the `controllo_tabella`. If we take as an example the `controllo_tabella` reported above (see description of phase E), the trajectory reported at z=3 by the H_track_3D.m routine corresponds to the target 612.

PHASE I: CONTINUITY OF THE FINAL TRAJECTORY (COMPILATION OF THE PARAGON TABLE).

I_CREATE_CROSS_MATRIX.M

This routine compiles the possible final trajectory (in the form of a table called `paragone`) composed of all the trajectories of the targets that appear in the `controllo_tabella`. The table is sorted in order of increasing passage times and the change in the x and the y position between subsequent rows of the table is computed and reported in the table for user inspection and help. An example of a possible output for the table paragon is given here.

Table I-1.

frame	x	y	target	Δx	Δy
1	193	204	246	-2	-15
2	191	189	547	1	14
2	192	203	246	-2	-12
4	190	191	546	3	12
4	193	203	246	-8	-6
5	185	197	244	1	-1
6	186	196	244	1	-1
7	187	195	544	5	9
7	192	204	246	-6	-7
9	186	197	244	0	5
12	186	202	862	1	-5
13	187	197	244	3	6
14	190	203	246	-3	-5
15	187	198	244	2	6
15	189	204	246	0	-1
17	189	203	246	0	-12

18	189	191	546	3	-2
19	192	189	547	-7	12
24	185	201	862	0	1
25	185	202	862	5	-11
25	190	191	546	-4	8
26	186	199	244	3	-7
26	189	192	546	-4	11
27	185	203	862	-1	-1
28	184	202	862	1	-2
29	185	200	244	0	0
29	185	200	862	5	4
31	190	204	246	-6	-4
33	184	200	244	0	0
33	184	200	862	3	-6
34	187	194	544	4	-4
36	191	190	547	-5	5
39	186	195	544	1	0
40	187	195	544	4	-6
40	191	189	547	-3	13
42	188	202	246	-1	-6
43	187	196	544	5	-6
43	192	190	547	-3	0
57	189	190	547	-5	9
60	184	199	244	0	0
60	184	199	862	5	-2
65	189	197	544	1	0
68	190	197	544	0	-1
71	190	196	544	2	1
72	192	197	544	-4	-4
73	188	193	546	0	0
73	188	193	547	6	3
73	194	196	544	1	-1
75	195	195	544	-1	0
76	194	195	544	1	1
79	195	196	544	-7	5
82	188	201	246	-1	1
83	187	202	246	9	-7
87	196	195	544	1	0
93	197	195	544	1	1
106	198	196	544	-9	-2
110	189	194	546	0	0
110	189	194	547	-4	5
116	185	199	244	0	0
116	185	199	546	0	0
116	185	199	547	0	0

116	185	199	862	11	-1
117	196	198	544	-9	5
119	187	203	244	0	0
119	187	203	246	0	0
119	187	203	546	0	0
119	187	203	547	0	0

From this table one can see that the table contains more than one trajectory. One possible choice of continuous trajectories obtained by the original table given above is for example:

Table I-2.

t	X	Y	Targ	Dx	Dy
34	187	194	544	-1	1
39	186	195	544	1	0
40	187	195	544	0	1
43	187	196	544	2	1
65	189	197	544	1	0
68	190	197	544	0	-1
71	190	196	544	2	1
72	192	197	544	2	-1
73	194	196	544	1	-1
75	195	195	544	-1	0
76	194	195	544	1	1
79	195	196	544	1	-1
87	196	195	544	1	0
93	197	195	544	1	1
106	198	196	544	-2	2
117	196	198	544		

t	X	Y	Targ	Dx	Dy
5	185	197	244	1	-1
6	186	196	244	1	-1
7	187	195	544	-1	2
9	186	197	244	1	0
13	187	197	244	0	1
15	187	198	244	-2	3
24	185	201	862	0	1
25	185	202	862	1	-3
26	186	199	244	-1	4
27	185	203	862	-1	-1
28	184	202	862	1	-2
29	185	200	244	-1	0
33	184	200	244	0	-1
60	184	199	862		

t	X	Y	Targ	Dx	Dy
12	186	202	862	3	2
15	189	204	246	0	-1
17	189	203	246	-1	-1
42	188	202	246	0	-1
82	188	201	246	-1	1
83	187	202	246		

t	X	Y	Targ	Dx	Dy
2	191	189	547	-1	2
4	190	191	546	-1	0
18	189	191	546	3	-2
19	192	189	547	-2	2
25	190	191	546	-1	1
26	189	192	546	2	-2
36	191	190	547	0	-1
40	191	189	547	1	1
43	192	190	547	-3	0
57	189	190	547	-1	3
73	188	193	546		

t	X	Y	Targ	Dx	Dy
1	193	204	246	-1	-1
2	192	203	246	1	0
4	193	203	246	-1	1
7	192	204	246	-2	-1
14	190	203	246	0	1
31	190	204	246		

In order to build the most complete paragon table for one target followed along the stack and to be fed to the phase K of the algorithm, the user should perform the phases E→I for all the targets that on the image appear to have possible interactions, and combine results similar to those shown in table I-2 obtained for different values of Punto_iniziale.

PHASE K: CREATION OF THE MOVIE OF THE FINAL TRAJECTORY.

K_PREPARE_PARAGON.M, K_FINAL_MOVIE.M AND K_BETA_FINAL_MOVIE_TRAJ.M

These routines help the user in building up a final trajectory movie and to compute the x,y trajectory to be used for further physical analysis. The K_final_movie.m and K_beta_final_movie_traj.m routines, that differ only for the display mode of the trajectory on the movie, take as an input the table `paragon`, created by the phase I of the algorithm and mount a movie with the trajectory superimposed on the image stack.

K_final_movie.m reports the trajectory as a collection of circles that follow the target in its motion.

K_final_movie_traj.m reports the trajectory as a trace that is superimposed on all the frames of the final movie.

The user must validate the table `paragon` before using these routines. The validation is performed by looking at the continuity of the trajectory on the table as described in the phase I. In order to perform this task the user can inspect visually the table `paragon`, as described in the phase I, or use the K_prepare_paragon.m routine.

K_prepare_paragon.m

The routine looks for the continuity in the x component of adjacent rows of the table paragon according to the parameter `threshold_paragon`.

The routine builds a matrix `trj(max_trj_build, max_len_trj,6)` in which at most max_trj_build sub-matrices extracted from the table `paragon`, according to the continuity test on the x coordinate of the trajectory, are stored sub-sequentially. The maximum length (number of elements) of the sub-trajectories is `max_len_trj`.

Since the visualization of a 3D matrix is not easy in the MATLAB editor, the routine provides, at each run, one of the continuous submatrices in a separate 2D matrix, `trj(max_len_trj, 6).`

Input:

`threshold_paragon`: value of the threshold for the continuity between adjacent rows in the table `paragon.` Default value is `threshold_paragon = 2;`

The algorithm with which the table **`paragon`** is split in continuous sub-matrices (locally stored as **`temp`** in the code excerpt reported below) is the following:

```
 indice1 = 1;
 indice2 = 1;
 i=1;
dim_par = size(paragone,1);
temp = paragone;

while ( (i<dim_par) && (temp(indice1,1)~=0))   % paragone last line is 0
    temp1(i,:) = temp(indice1,:);
    j = min(indice1 + 1,dim_par);
    scarto = abs(temp(j,2)-temp1(i,2));
```

```
     while ((j<dim_par)&&(scarto > threshold_paragon))
        temp2(indice2,:) = temp(j,:);
        indice2=indice2+1;
        j= j+1;
        scarto = abs(temp(j,2)-temp1(i,2));
     end
    indice1 = j;
    i = i+1; % back again
 end
```

max_trj_build: is the maximum number of continuous sub-matrices to be built. The default value is max_trj_build =8.

out_trj: is the index number of the sub-trajectory to be stored in the output 2D matrix trj_cur(max_len_trj,6) for the user inspection. Default value is out_trj = 1. This parameter should be changed by the user before running the routine in order to obtain a readable 2D matrix to be used to run K_final_movie.m or K_final_movie_traj.m. One needs only to replace the matrix paragone with one of the trj_cur output matrices obtained by this routine (K_prepare_paragon.m).

max_len_trj. Is the maximum length of the sub-trajectories to be built. Default value is max_len_trj = 200;

Output:

trj_cur. This is a 2D matrix that stores a subset of the table paragon that satisfies the continuity constraint. This table represents the projection of the trj(max_trj_build,max_len_trj,6) on one of the indices (chosen by setting the index out_trj before running the routine) of the first dimension:

```
trj_cur( : , : )  = trj(out_trj, : , : )
```

The trj_cur matrix has the same dimension (paragone(max_len_trj,6)) as paragone and can therefore be used as it is to replace paragone before running K_final_movie.m or K_final_movie_traj.m. The whole sets of continuous sub-trajectories is stored in trj(max_trj_build, max_len_trj,6). In order to get all the subtrajctories in a format suitable for further use in the K phase one should re-run K_prepare_paragon.m with different values of the out_trj parameter.

K_final_movie.m and K_beta_final_movie_traj.m

Input:

sogliaalta: is the value of a threshold to be used to enhance the contrast in the image. Default value is 10.

Trajectory coding.

K_final_movie.m: In this routine the image is coded in jet colormap and the position of the target is marked by a moving white circle in the image.

K_beta_final_movie_traj.m:In this routine the image can be coded in jet or gray colormap (direct or inverted) and the trajectory is marked as a fixed collection of pixels in which the occupancy time is encoded (replacing the image colormap) in white, jet or gray colormaps. These different options can be chosen by the user by setting the parameter display_bg. Default value is display_bg = 0;

The coding is performed as it follows:

```
%% if 0 gray inverted image with increasing green level coding of the traj
                %% if 1 jet image with white traj
                %% if 2 gray image with jet traj
                %% if 3 gray inverted image with gray normal lut for the traj
```

Output:

The movie is mounted by copying the frames initially displayed on the screen on an avi file, whose name is created as TEST”Punto_iniziale”.avi'. For example if the target whose trajectory is being analyzed is Punto_iniziale=655, the name of the file in the current working directory will be “TEST655.avi”.

The Movie is also opened on the screen by means of the Matlab encoded avi player, implay.

Important notes on the color coding and the numbering of the starting pixels of the trajectory on the image displayed by the D_add_label.m routine.

Let us take first a reference image. This is taken from the stack of images reporting the motion of the SLN nanoparticles within the cells, reported in Fig.2 and discussed in the text.

The image reported below corresponds to the bottom row of images in Fig.2. We first run the D_add_label.m routine with typical parameters.

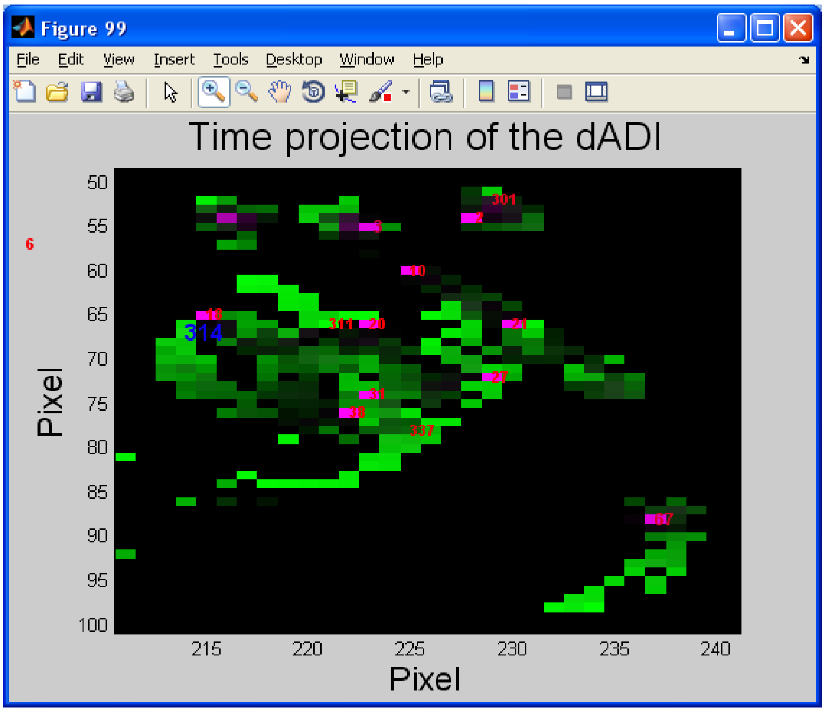

Parameters.

File:scan1_n120_s210.mat.

```
numero = 120,
threshold = 0.0210.
```

For C_coord_Vector.m:

```
lunghezza=6;
incrementostep=119;
```

For D_beta_add__label_beta.m:

```
length_traj=6;
T_F_size_0 = 10;
D_T_F_size = 2
```

D_beta _add_label.m implements the possibility to choose the text font size. We could also give different sizes of the numbers reported in the image since the size is chosen according to two parameters

```
T_F_size_0 = 10;  % initial font size
D_T_F_size = 0;  % increment on the font size
```

and the following algorithm:

```
T_F_size = T_F_size_0 + D_T_F_size*fine(1,1,i)/colore;
```

where *fine(1,1,i)* is the occupancy time of the pixel and the variable *colore* reports the maximum value of the occupancy time for the trajectory.

Color map of the trajectory.

Let us consider first the issues related to the choice of the colors of the trajectory pixels. This is a green colormap as reported in the Figures. However we also encode the fact that a trajectory starts from the very first frames of the stack, by coloring magenta that pixel.

Example 1 within the square (Dx,Dy)=(210:243,50:100) approx.

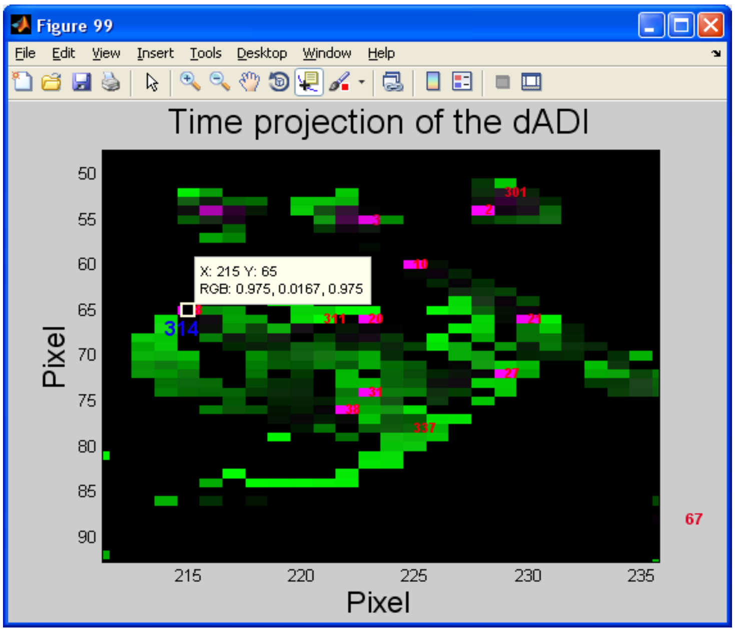

Take the magenta pixel numbered "18". This corresponds to the 18th trajectory. It corresponds to the pixel (215,65) with RGB values (output of the D_add_label.m) (R,G,B) = (0.975,0.0167,0.975). By debugging the run we get that this pixel corresponds to the starting point of a trajectory very long (it is mapped on 116 out of 120 frames total) and starts from the very first frame.

The algorithm for building up the colors in the trajectory is implemented in D_add_label.m as follows:

```
for i=1:g-1,
    for k=1: 256
        for j=1: 256
            if abs(bw3(k,j,1)-bw3(k,j,i+1))>0.5,
                Htempo3(k,j,1)=Htempo3(k,j,1)+1;
                if Htempo3(k,j,2)==0, Htempo3(k,j,2)=Htempo3(k,j,2)+i;end;
                Htempo3(k,j,3)=Htempo3(k,j,3)+1;
            end
        end
    end
end;
```

The `Htempo3(x,y,:)` matrix contains the RGB components of the (x,y) pixel. This matrix contains, at the end of the run, a picture of the motion. The piece of code reported above, instructs the program to assign to the G component (i.e. `Htempo(x,y,2))` of the (x,y) pixel the value of that frame ("i" in the code) on which some motion has been detected for the first time on the specified pixel. The R and B components (`Htempo(x,y,1)` and `Htempo(x,y,3)`) are continuously

incremented each time that the pixel has been interested by a motion. So, at the end, these two values report the number of times we have detected a motion on the specified pixel.
Therefore those long trajectories that starts from the first frames corresponds to RGB triples in which the G value is much less than the R and B ones, and result in a magenta color.

Example 2 within the square (Dx,Dy)=(210:243,50:100) approx.

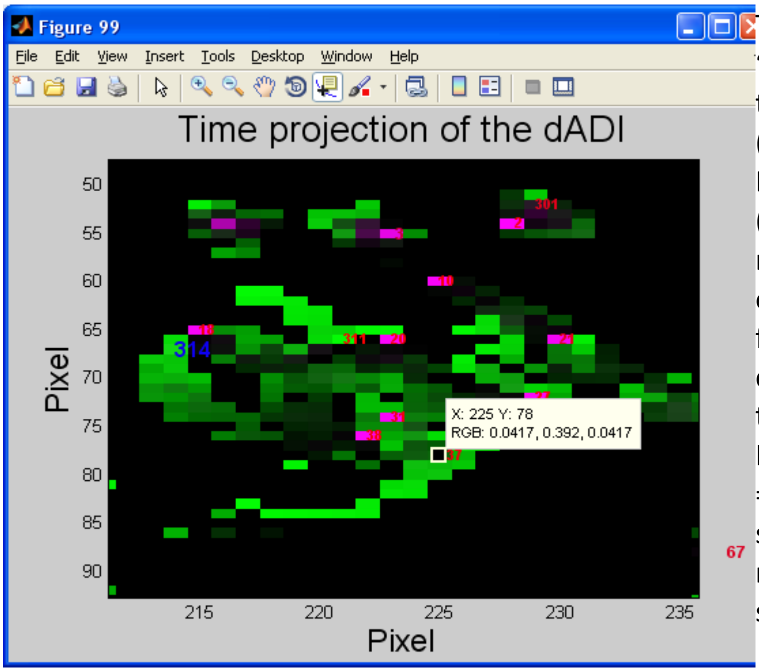

Take the dark green pixel numbered "337". This corresponds to the 337th trajectory. It corresponds to the pixel (225,78) with RGB values (output of the D_add_label.m) (R,G,B) = (0.0417,0.392,0.0417). By debugging the run we get that on this pixel a change occurred for the first time on the 47th frame and that only on 5 frames total a change occurred on this pixel. Therefore the Htempo3(225,78,2)= 47 and Htempo3(225,78,1)=Htempo3(225,78,3) =5. In such a case the R and B values are small (0.0417) and the G value is relatively large (0.392) but definitely smaller than one.

Example 3 within the square (Dx,Dy)=(210:243,50:100) approx.

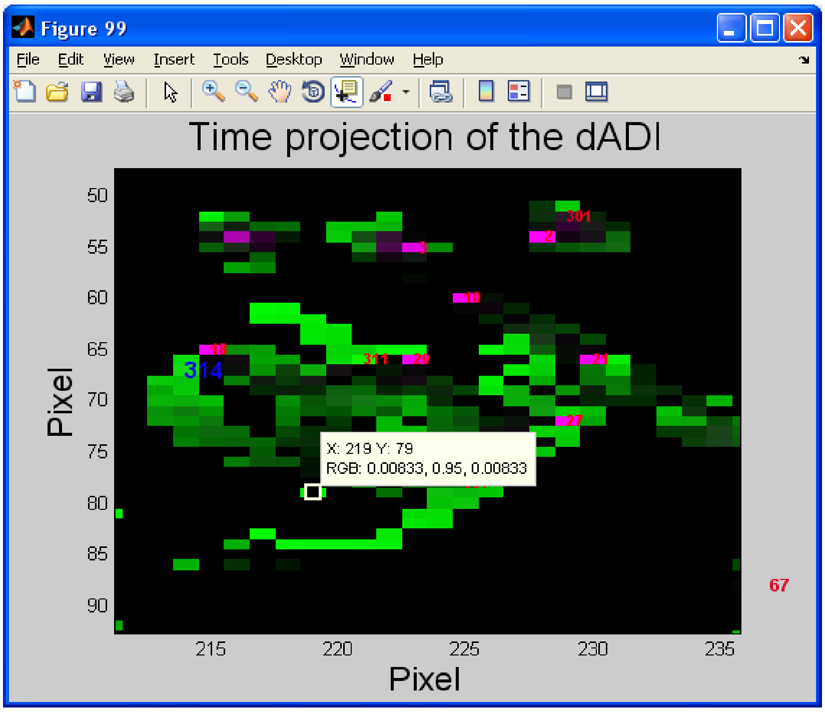

Take the pixel (219,78). This pixel does not correspond to the starting position of any trajectory: its position does not correspond to (x,y) = (fine(1,3,i), fine(1,2,i)) for any value of i=1..punto_tot (punto_tot is the total number of trajectories).
The content of the image on this pixel corresponds to RGB values (output of the D_add_label.m) (R,G,B) = (0.00833,0.95,0.00833).

By debugging the run we get that on this pixel a change occurred for the first time on the 114th frame (out of 120 analyzed) and that only on 1 frame a change occurred on this pixel. Therefore

the Htempo3(225,78,2)= 114 and Htempo3(225,78,1)= Htempo3(225,78,3) = 1. In such a case the R and B values are small (0.00833) and the G value is very large (0.95), so that the pixel is bright.

Positioning of the text on the image.

Regarding the position of the target number on the output image of D_add_label.m we note that the number is always set in such a way that the most significant digit lies at the center of the pixel and the other digits are then found on the right side of the pixel. As an example of this algorithm we report the following image:

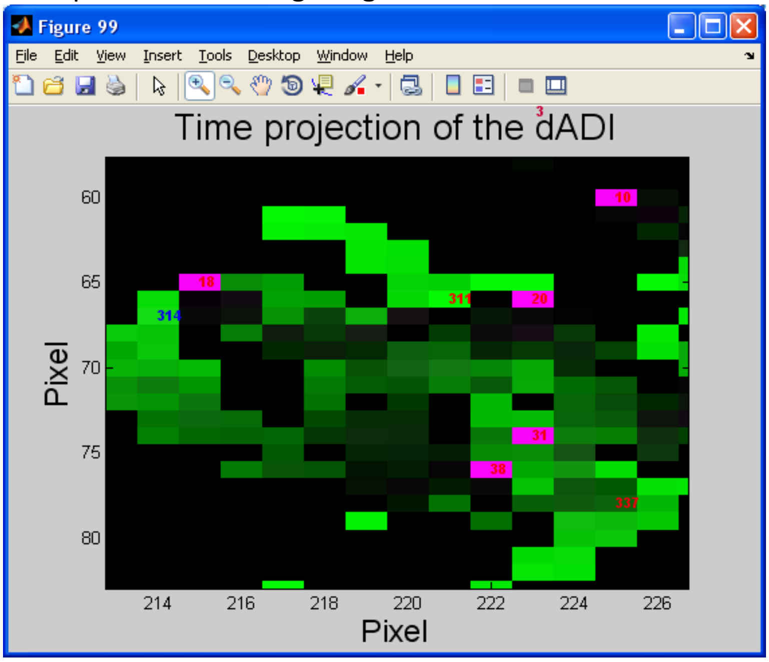

A specific parameter (`T_F_size=12`) in the D_add_label routine specifies for the font size. It may help to lower the value of this parameter to avoid the partial superposition of targets' number on the image.

For example if we run D_add_label with

```
length_traj=2;
T_F_size_0 = 12;
```

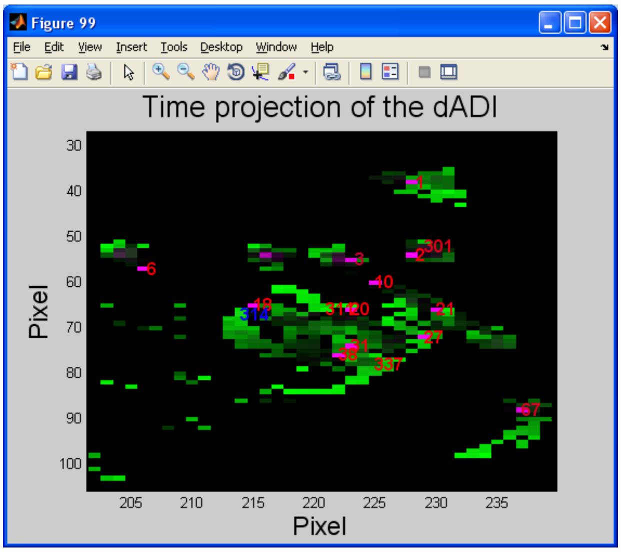

We obtain the following (zoomed) image on which some of the targets' number are superimposed. This is due to the large font size and the high density of targets due to the low constraints imposed by the algorithm (particularly low value of the threshold on the length of the trajectory to be displayed, length_traj=2).

If on the contrary we run the D_add_label with a lower font size, We obtain for the same area of the image the following result in which no superposition of targets' indices can be found.

For example if we run D_add_label with

```
length_traj=2;
T_F_size_0 = 8;
```

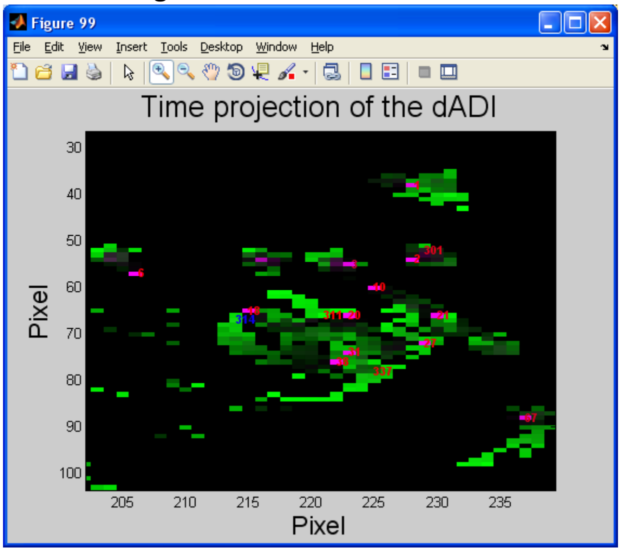

In addition to the choice of the font size we have now added to the D_add_label.m routine a feature that check for possible real superposition of the numbers on the image. This may occur for entangled trajectories, since the same pixel may belong to more than one trajectory and may be tracked from different initial frames (if incrementostep is chosen less than the total number of frames). If a superposition occurs the routine displays by one unit in the nearest neighbors the text reporting the target number and uses for this text the white color, in order to notify the user of this instance.

It is very important to notice that Matlab does not scale the size of the text on an image when the user zoom in or out the image itself. The location of the numbers with respect to starting pixel of the trajectory depends then on the zoom of the image. However, by zooming in the image the location of the numbers with respect to the starting pixels becomes more and more accurate, as can be gained by the following sequence of images taken at increasing zoom level (see also the animated gif image: AnimatedZoom_forTextPositioning.gif as OAM).

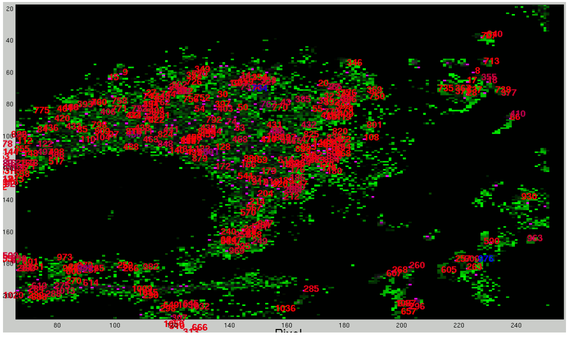

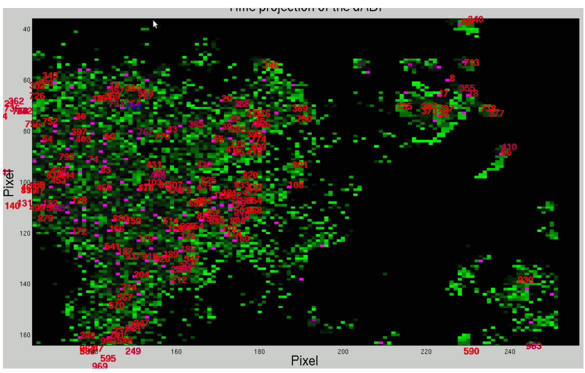
Pixel
Pixel

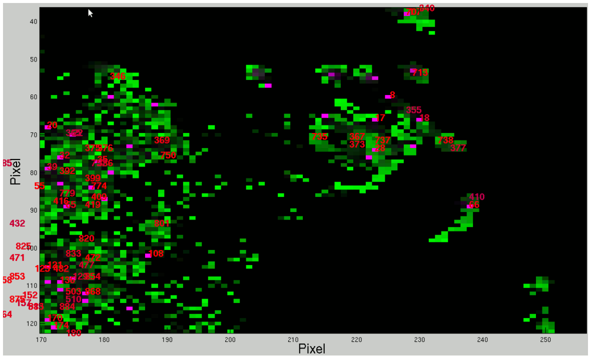
Pixel
Pixel

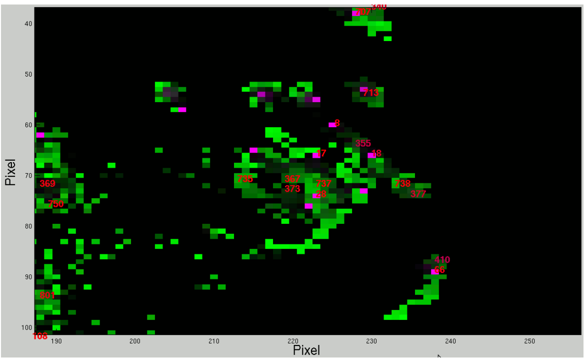
Pixel
Pixel

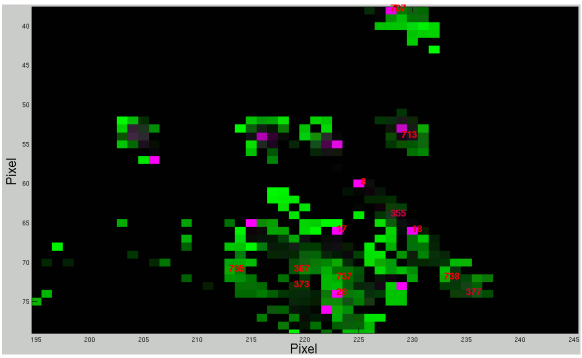
Pixel
Pixel

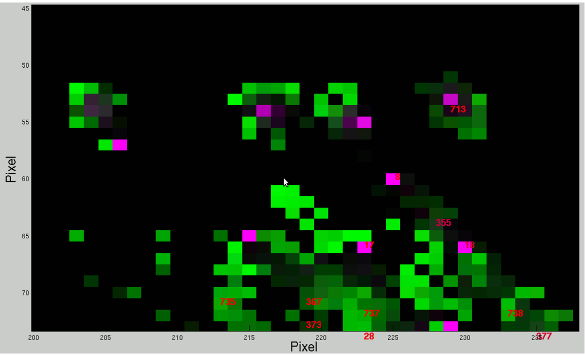

Pixel
45
50
55
60
65
70
200
205
210
215
220
225
230
Pixel
713
8
355
17
18
735
367
737
738
373
28
377

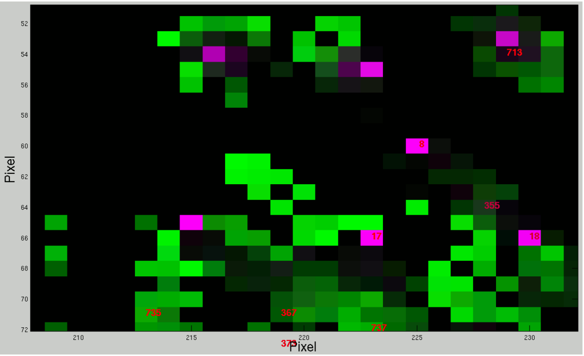

Pixel
52
54
56
58
60
62
64
66
68
70
72
210
215
220
225
230
Pixel
713
8
355
17
18
735
367
737
