# Supplementary figures and images for: Accumulative Difference Image Protocol for Particle Tracking in Fluorescence Microscopy Tested in Mouse Lymphonodes
Source: PLoS One. 2010 Aug 17;5(8):e12216. doi: 10.1371/journal.pone.0012216 (PMC2923183; doi:10.1371/journal.pone.0012216)

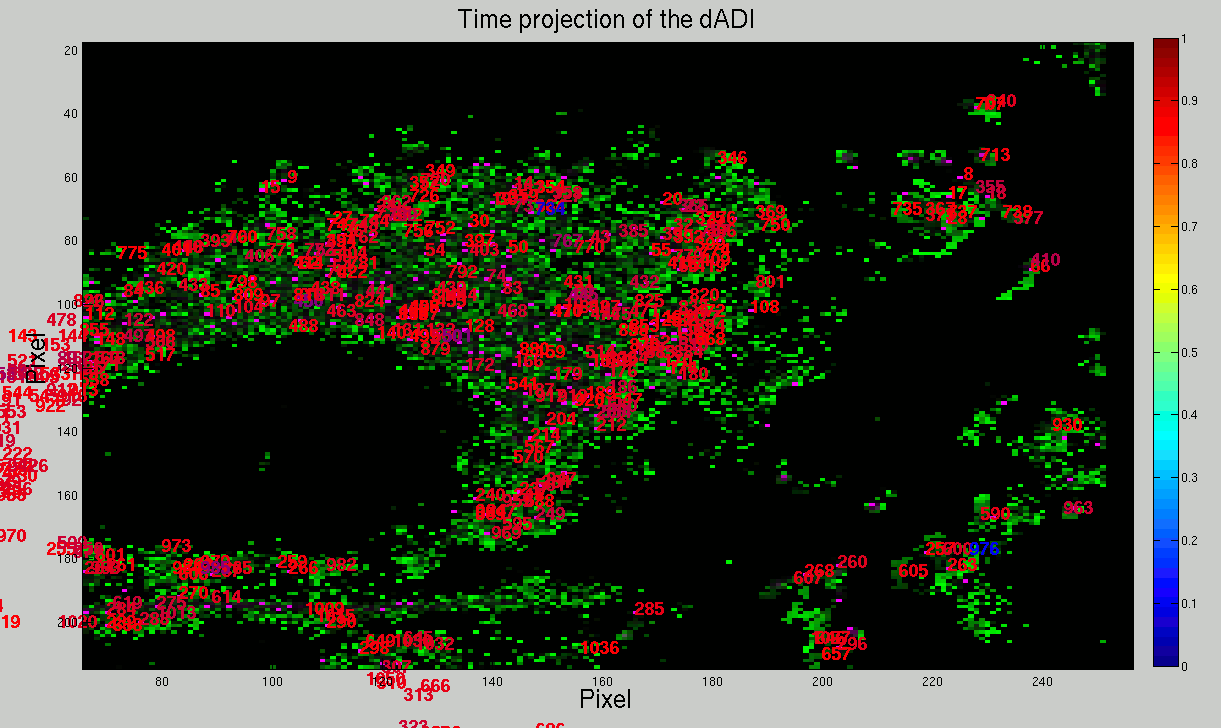

Supplement: Movie S2 — This is an animated GIF file that visualize the effect, on the positioning of the number of the specific target, of zooming into an image with MATLAB. (0.60 MB GIF) [file pone.0012216.s004.gif]
